# *Clostridium difficile* exosporium cysteine-rich proteins are essential for the morphogenesis of the exosporium layer, spore resistance, and affect *C*. *difficile* pathogenesis

**DOI:** 10.1371/journal.ppat.1007199

**Published:** 2018-08-08

**Authors:** Paulina Calderón-Romero, Pablo Castro-Córdova, Rodrigo Reyes-Ramírez, Mauro Milano-Céspedes, Enzo Guerrero-Araya, Marjorie Pizarro-Guajardo, Valeria Olguín-Araneda, Fernando Gil, Daniel Paredes-Sabja

**Affiliations:** 1 Microbiota-Host Interactions and Clostridia Research Group, Departamento de Ciencias Biológicas, Facultad de Ciencias de la Vida, Universidad Andrés Bello, Santiago, Chile; 2 Millennium Nucleus in the Biology of the Intestinal Microbiota, Facultad de Ciencias de la Vida, Universidad Andrés Bello, Santiago, Chile; University of Texas Medical School at Houston, UNITED STATES

## Abstract

*Clostridium difficile* is a Gram-positive spore-former bacterium and the leading cause of nosocomial antibiotic-associated diarrhea that can culminate in fatal colitis. During the infection, *C*. *difficile* produces metabolically dormant spores, which persist in the host and can cause recurrence of the infection. The surface of *C*. *difficile* spores seems to be the key in spore-host interactions and persistence. The proteome of the outermost exosporium layer of *C*. *difficile* spores has been determined, identifying two cysteine-rich exosporium proteins, CdeC and CdeM. In this work, we explore the contribution of both cysteine-rich proteins in exosporium integrity, spore biology and pathogenesis. Using targeted mutagenesis coupled with transmission electron microscopy we demonstrate that both cysteine rich proteins, CdeC and CdeM, are morphogenetic factors of the exosporium layer of *C*. *difficile* spores. Notably, *cdeC*, but not *cdeM* spores, exhibited defective spore coat, and were more sensitive to ethanol, heat and phagocytic cells. In a healthy colonic mucosa (mouse ileal loop assay), *cdeC* and *cdeM* spore adherence was lower than that of wild-type spores; while in a mouse model of recurrence of the disease, *cdeC* mutant exhibited an increased infection and persistence during recurrence. In a competitive infection mouse model, *cdeC* mutant had increased fitness over wild-type. Through complementation analysis with FLAG fusion of known exosporium and coat proteins, we demonstrate that CdeC and CdeM are required for the recruitment of several exosporium proteins to the surface of *C*. *difficile* spores. CdeC appears to be conserved exclusively in related Peptostreptococcaeace family members, while CdeM is unique to *C*. *difficile*. Our results sheds light on how CdeC and CdeM affect the biology of *C*. *difficile* spores and the assembly of the exosporium layer and, demonstrate that CdeC affect *C*. *difficile* pathogenesis.

## Author summary

We discovered a mechanism of assembly of the outer most layer of Clostridium difficile spores, the exosporium. While CdeC is conserved in several Peptostreptococcaeace family members, CdeM is unique to C. difficile. We show that two proteins that are rich in cysteine amino acid residues, CdeC and CdeM, are essential for the recruitment of additional spore coat and exosporium proteins. The absence of CdeC, had profound implications in the correct spore coat assembly which were related to decreased spore resistant properties that are relevant for *in vivo* infection such as lysozyme resistance, macrophage infection. Notably, the absence of either cysteine rich proteins leads to a decrease in spore adherence of C. difficile spores to healthy colonic mucosa; but only the absence of CdeC affected *in vivo* competitive fitness in a mouse model, recurrence of the disease in a mouse model of recurrent infection. Considering the importance of the outer layers of C. difficile spores in spore-host interactions, our findings have broad implications on the biology of C. difficile spores and to C. difficile pathogenesis.

## Introduction

*Clostridium difficile* [1], first reclassified as *Peptoclostridium difficile* [1] and more recently re-classified as *Clostridioides difficile* [2], is a Gram-positive, sporogenic anaerobic bacterium that is the most common cause of antibiotic-associated diarrhea within healthcare systems of the developed world [3, 4]. The clinical manifestation of the infection is diarrhea and in severe cases can produce pseudomembranous colitis, toxic megacolon and death [5]. Mortality of *C*. *difficile* infections (CDI) may reach up to 5% of CDI cases, but in several outbreaks, it has increased up to 20% [3]. Conventional metronidazole and/or vancomycin treatment (depending on the severity of the symptoms) although resolve single episodes of CDI, exhibit high rates of recurrence of the infection after a first episode. The rate of recurrence of CDI of a first, second and third episode may reach up to 20%, 40% and 60%, respectively [6, 7].

During the infection, *C*. *difficile* colonization leads to secretion of large toxins (TcdA and TcdB) that glycosylated intestinal epithelial cell proteins, induce massive inflammation of the gut epithelium, causing disease symptoms ranging from mild diarrhea to pseudomembranous colitis, toxic megacolon and even death [8]. However, before *C*. *difficile* can colonize a susceptible host, the highly resistant and metabolically dormant spore must germinate in response to secondary bile salts present in high levels in the gastrointestinal tract of antibiotic-treated host [9, 10]. In addition to toxin-production during *C*. *difficile* colonization of the host, a subset of *C*. *difficile* vegetative cells initiates a sporulation program that culminates with the formation of metabolically dormant spores [11, 12]. These spores have intrinsic resistance properties enabling their survival to enzymatic degradation [13, 14], phagocytic cells [15] and chemicals normally found in the host´s gastrointestinal (GI) environment [16], enabling their persistence in the host´s GI tract.

To persist in the host, *C*. *difficile* spores must interact with the host´s colonic mucosa through specific interactions mediated by spore-ligand(s) molecules and host cellular receptor(s) [17]. In this context, as demonstrated in other spore-former species [18], the surface of *C*. *difficile* spores is likely to be the primary site of spore-host interactions that contributes to spore persistence. Consequently, there is keen interest to understand fundamental aspects of the outermost exosporium layer of *C*. *difficile* spores [19]. Notably, the exosporium layer of *C*. *difficile* spores differs from previously described outermost layers [19–21]. For example, in contrast with the exosporium layer of spores of the *Bacillus cereus* group, where an interspace gap separates the exosporium from the spore coat [18, 19, 22], the exosporium of *C*. *difficile* spores is in direct contact with the spore coat layers in a similar fashion as the outer crust of *Bacillus subtilis* spores [13, 18, 19]. Despite these differences with the outer layer of spores of other bacterial endospore formers, the exosporium layer of most *C*. *difficile* strains have hair-like extensions similarly as those observed in spores of the *B*. *cereus* group [19, 22]. However, in striking difference from other endospore formers, during the sporulation program, *C*. *difficile* forms spores with two distinctive exosporium morphotypes that arise from the same clonal sporulating culture, during either standard sporulation conditions (i.e., agar plates), or during biofilm development conditions [20, 21]. These exosporium morphotypes include: i) spores with a thick-exosporium layer, defined by an electron dense material surrounding the spore coats; and ii) a thin-exosporium layer, where the electron-dense material that surrounds the spore coat is notably thinner [20, 21].

Recently, the composition of the outermost exosporium layer of *C*. *difficile* spores of the laboratory 630*erm* strain has been determined with several interesting features [23]. Orthologs of the BclA family of proteins have been identified, yet the structural proteins known to be involved in the exosporium assembly of the exosporium layer of the *B*. *cereus* group, are absent in the *C*. *difficile* exosporium proteome [23]. Moreover, CdeC, CdeM, CdeA and CdeB were shown to be uniquely localized in the exosporium layer of *C*. *difficile* 630*erm* spores and accessible to antibodies [23], suggesting exposure to the spore-surface; of these, CdeC and CdeM exhibited an unusually high content of cysteine residues [23]. Cysteine-rich proteins have been reported to be essential for the assembly of the exosporium in *B*. *anthracis* spores (i.e., ExsY) [24, 25] and of the outer crust layer in *B*. *subtilis* spores (i.e., CotY and CotZ) [26]. In *B*. *subtilis*, the cysteine-rich proteins of the spore crust, CotY and CotZ, are capable of cooperatively self-assembling into thermally stable structures favored by strong disulfide cross-linking [27].

Studies on the outer spore layer of *C*. *difficile* have shown that 630*erm* strain forms spores that albeit have both exosporium morphotypes, they lack the hair-like projections observed in most epidemic strains [19–21], suggesting that the mechanisms underlying exosporium assembly might exhibit slight difference between both strains. For example, the cysteine-rich protein, CdeC, shown to be required for the morphogenesis of the coat and exosporium layer of spores of the epidemically relevant R20291 strain [13, 19], is present at 100-fold higher levels in 630*erm* spores compared to R20291 spores [23] and exhibits a deletion in the N-terminal domain (S3 Fig). The only known functional role of CdeM is that inactivation of *cdeM* leads to a loss of competitive fitness during infection of germ free mice [11]. Consequently, it is likely that both cysteine-rich proteins, CdeC and CdeM, might be involved in the differences observed between the exosporium layer of 630*erm* and R20291 spores. In this context, in our systematic approach to gain more insight into the mechanisms of assembly of the exosporium layer of *C*. *difficile* spores, the aim of this work was to address the functional roles of CdeC (i.e., CD1067 in 630*erm* strain) and a novel morphogenetic factor, CdeM (CD1581 in strain 630*erm*). Using a series of microscopic, genetic, molecular biology and cellular biology assays, we have characterized the *cdeC* and *cdeM* phenotypes and demonstrate their implications in the assembly of the exosporium layer and *C*. *difficile* spore biology. We also demonstrate that the absence of CdeC and CdeM differentially affect *in vivo* spore adherence, infection recurrence, and fitness in a series of mouse models, contributing to understand their implications in *C*. *difficile* pathogenesis.

## Results

### *In silico* analysis of two cysteine rich proteins, CdeC and CdeM

**Fig 1:**
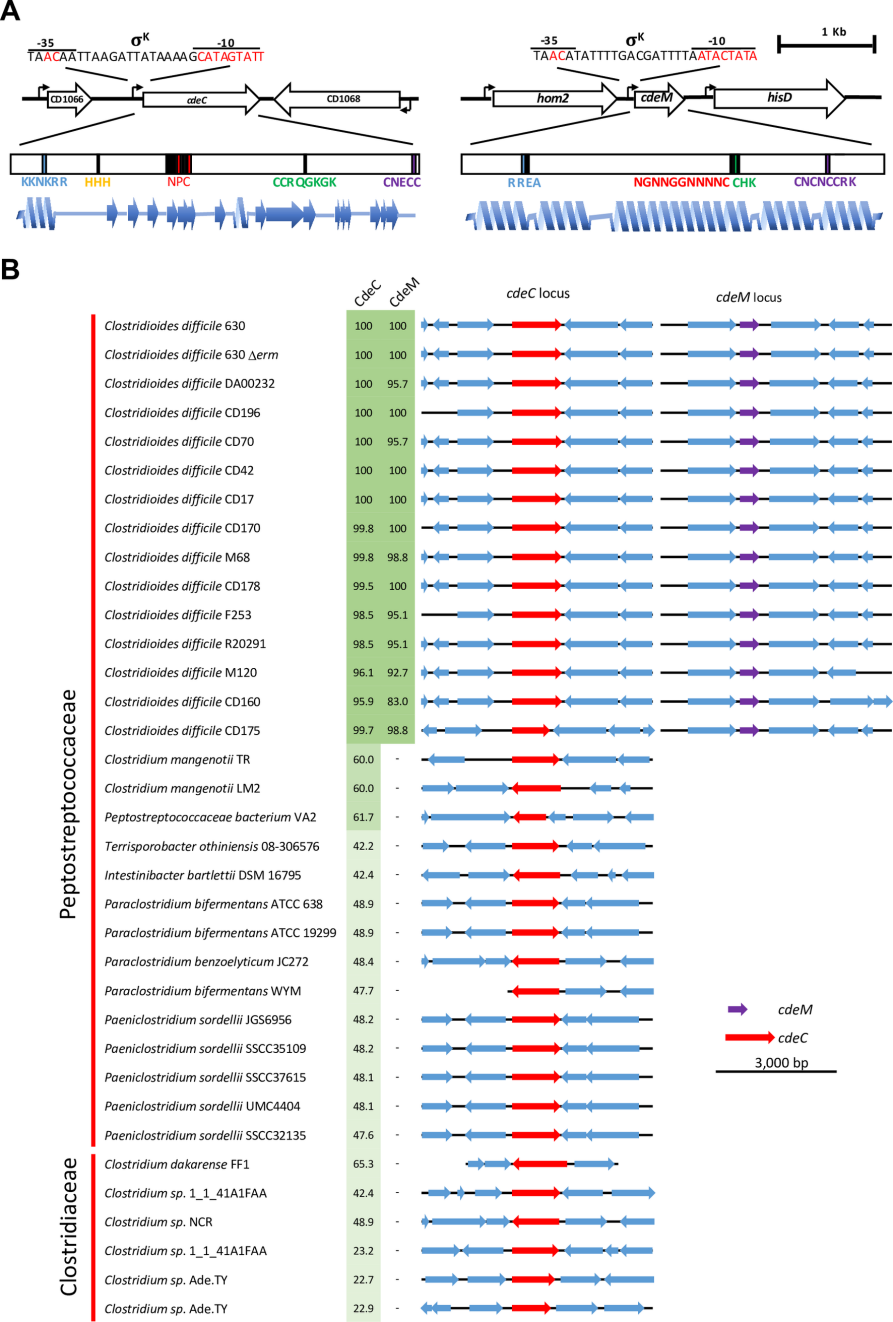
Schematic representation of CdeC and CdeM proteins and their conservation in *C*. *difficile* and other Peptostreptococcaceae family members. **(A)** *cdeC* is found downstream of *CD1066*, which encodes a putative protein of unknown function protein and upstream of *CD1068* which is an antisense encoding ORF of a putative protein involved in polysaccharide biosynthesis, and expressed during sporulation protein encoded in the antisense complementary sequence; all three are monocistronic genes. Notably, a putative σ^K^-regulated promoter is located immediately upstream of *cdeC*, whose position was mapped by RNA-Seq [28] (shown in the scheme). By contrast, *CD1066* and *CD1068* have putative σ^E^-regulated promoter immediately upstream of their ORFs [28, 30]. By contrast, *cdeM* is found downstream of *CD1580* which encodes a putative homoserine dehydrogenase (Hom2), and upstream of *CD1582* encoding a putative histodinidol dehydrogenase (HisD). Transcription of *cdeM* is predicted (by RNA-Seq) [28] to be under the control of a σ^K^-regulated promoter immediately upstream of *cdeC*; however, transcription of *hom2* and *hisD* is not dependent on sporulation [28, 30]. The main repeats in CdeC and CdeM are shown in the magnification of the predicted protein primary sequence in color and described in the text. Blue spirals and arrows indicate the predicted beta sheets and alpha helixes [31, 32]. **(B)**, Gene neighborhoods of predicted-coding regions whose products have homology to *C*. *difficile* 630*erm* CdeC and CdeM by a blastp search. The diagram is abridged to show only the first neighborhood in each genome for the *cdeC* locus and *cdeM* locus. Predicted proteins with homology to *C*. *difficile* CdeC and CdeM were clustered by sequence identity (S2 and S3 Figs). The percentage of identity with *C*. *difficile* 630*erm* CdeC and CdeM of homologues in other species is shown in green.

A recent proteomic study [23] identified two cysteine rich proteins (i.e., CdeC [CD1067] and CdeM [CD1581]) which were uniquely located in the outermost exosporium layer of 630*erm* spores [23]. Functional analysis of CdeC in the epidemic R20291 strain demonstrated that this protein is required for the correct assembly of the exosporium layer of R20291 spores [13]. However, the higher levels of CdeC observed in 630*erm* spores, suggests that CdeC might have a more predominant role in the assembly of the exosporium layer in 630*erm* spores, while the role of CdeM remains unclear. Both proteins are encoded by monocistronic genes whose promoters are controlled by the late-mother cells specific sigma factor, σ^K^ (Fig 1A), which the late-mother cells specific [28, 29]. *cdeC* in 630*erm* is flanked by genes encoding uncharacterized proteins transcribed by σ^E^-regulated promoters; by contrast, *cdeM*, located 570,775 bp downstream of *cdeC*, is flanked by genes encoding enzymes involved in amino acid biosynthesis (Fig 1A).

The 1218-bp *cdeC* gene encodes a 405-amino acid protein with a predicted molecular weight of 44.7-kDa, and a high content of cysteine residues (9% of the amino acid content), suggesting that it might be prone to disulfide bridge formation and therefore, play a role in the crosslinking of other exosporium proteins [27] [26]. Analysis of the amino acid sequence revealed no conserved domains, but several noteworthy sequence repeats conserved in all sequenced genomes of *C*. *difficile*: i) in the N-terminal domain (NTD) two motifs of unknown function were identified (i.e., KKNKRR and three consecutive histidine residues); ii) a 3xHistidine repeat near the NTD; iii) in the central region, a 6 NPC repeat followed by two CCRQGKGK repeat; and iv) cysteine rich sequence CNECC at the C-terminal domain (CTD) of CdeC (Fig 1A).

The 483-bp *cdeM* gene encodes a 161-amino acid encoded protein with a predicted molecular weight of 19.1-kDa, and a high content of cysteine residues (8.7% of the amino acid sequence), suggesting that CdeM, similarly as CdeC, might also be prone to disulfide bridge formation contributing to the crosslinking of exosporium proteins. Analysis of the primary sequence of CdeM gave no conserved domains, but some interesting features: i) three RREA repeats near the NTD of CdeM; ii) two NGNNGGNNNNC and three CHK repeats in the central region of CdeM; and iii) two CNCCNCCRK repeats at the CTD (Fig 1A).

### The CdeC and CdeM cysteine rich proteins are highly conserved in Peptostreptococcaceae family members, while CdeM is unique to *C*. *difficile*

Since we observed unique sequences in these two proteins, we wondered how conserved the CdeC and CdeM was among other *C*. *difficile* and related Peptostreptococcaceae family members, due to a recent reclassification of *C*. *difficile* into the *Clostridioides* genus, a member of the Peptostreptococcaceae family rather than in the Clostridiaceae family [1]. To assess the conservation of the cysteine rich proteins, CdeC and CdeM, in other Clostridial organisms, we searched for protein homologues to the *C*. *difficile* CdeC and CdeM in a blastp search (Fig 1B).

This analysis was performed in a chosen subset of strains of a wide variety of ribotypes and *C*. *difficile* genome groups (S2 Table, S1 Fig); both, *cdeC* and *cdeM*, were found to be conserved in all *C*. *difficile* isolates tested (Fig 1B. Interestingly both, CdeC and CdeM, and their unique repeats were present in all *C*. *difficile* strains analyzed (S2 and S3 Figs). Three out of 15 strains encoded a CdeC with a truncated NTD (S2 Fig), while five out of 15 *C*. *difficile* strains had an insertion in the NTD of CdeM (S3 Fig). Taken together, CdeC and CdeM are highly conserved in *C*. *difficile* representative strains.

When a blastp against *C*. *difficile* CdeC and CdeM was expanded to include additional members of the Peptostreptococcaceae, we observed that CdeC was conserved in all 8 Peptostreptococcaceae family members analyzed (Figs 1B and S2, S2, S3 and S4 Tables). By contrast, CdeM was unique to *C*. *difficile* (Figs 1B, S2, S3 and S4). Notably, despite the absence of CdeM, the genomes of *Clostridioides mangenotii*, *Paraclostridium bifermentas*, *Paraclostridium sordellii*, *Peptostreptococcaceae bacterium*, *Terrisporobacter othiniensis*, *Paraclostridium benzolyticum* had different CdeC variants with most of the sequence motifs conserved (Figs 1B, S2, S3 and S4). These results collectively suggest that, while CdeM is specific for *C*. *difficile*, CdeC is a conserved exosporium protein in members of the Peptostreptococcaceae family.

We sought to apply a similar analysis to a subset of Clostridiaceae and Lachnospiraceae family members to evaluate whether *C*. *difficile* CdeC and CdeM were present (S4 and S5 Tables). Strikingly, only CdeC but not CdeM, was found in members of the Clostridiaceae family, specifically in *Clostridium dakarense* and 5 *Clostridium sp*. (Fig 1B and S4 and S5 Tables). Despite the phylogenetic divergence (S5 Fig), the cysteine residues in the conserved motifs of CdeC are highly conserved in members of the Peptostreptococcaceae and Clostridiaceae families (S6 and S7 Figs). CdeC and CdeM were not present in members of the Lachnospiraceae family. Collectively, these results indicate that although CdeC is present in a few members of the Clostridiaceae family, the amino acid sequence is highly conserved in them.

### Construction of *cdeC* and *cdeM* mutant strains in a 630*erm* background

To evaluate the functional role of CdeC and CdeM in *C*. *difficile* 630*erm* strain, we used ClosTron mutagenesis by redirecting the group II L1.ltrB intron into the antisense strands of the N-terminal domain of both genes at positions 30 and 123 to inactivate *cdeC* and *cdeM*, respectively (S8A, S8B and S8C Fig). After many attempts to inactivate each individual gene, we were able to obtain several independent mutant clones of *cdeC* and *cdeM* as shown by PCR screening for insertions (S8A and S8B Fig) [33]. Mutants were confirmed by PCR using flanking primers and sequencing of the PCR amplicons (S8A and S8B Fig). Clones C2, C4 and C8 for *cdeC* mutant strain and C2, C3 and C4 for the *cdeM* mutant strain. These clones were used for further phenotypic characterization.

### CdeC and CdeM cysteine rich proteins are essential for the morphogenesis of the exosporium layer of *C*. *difficile* spores

**Fig 2:**
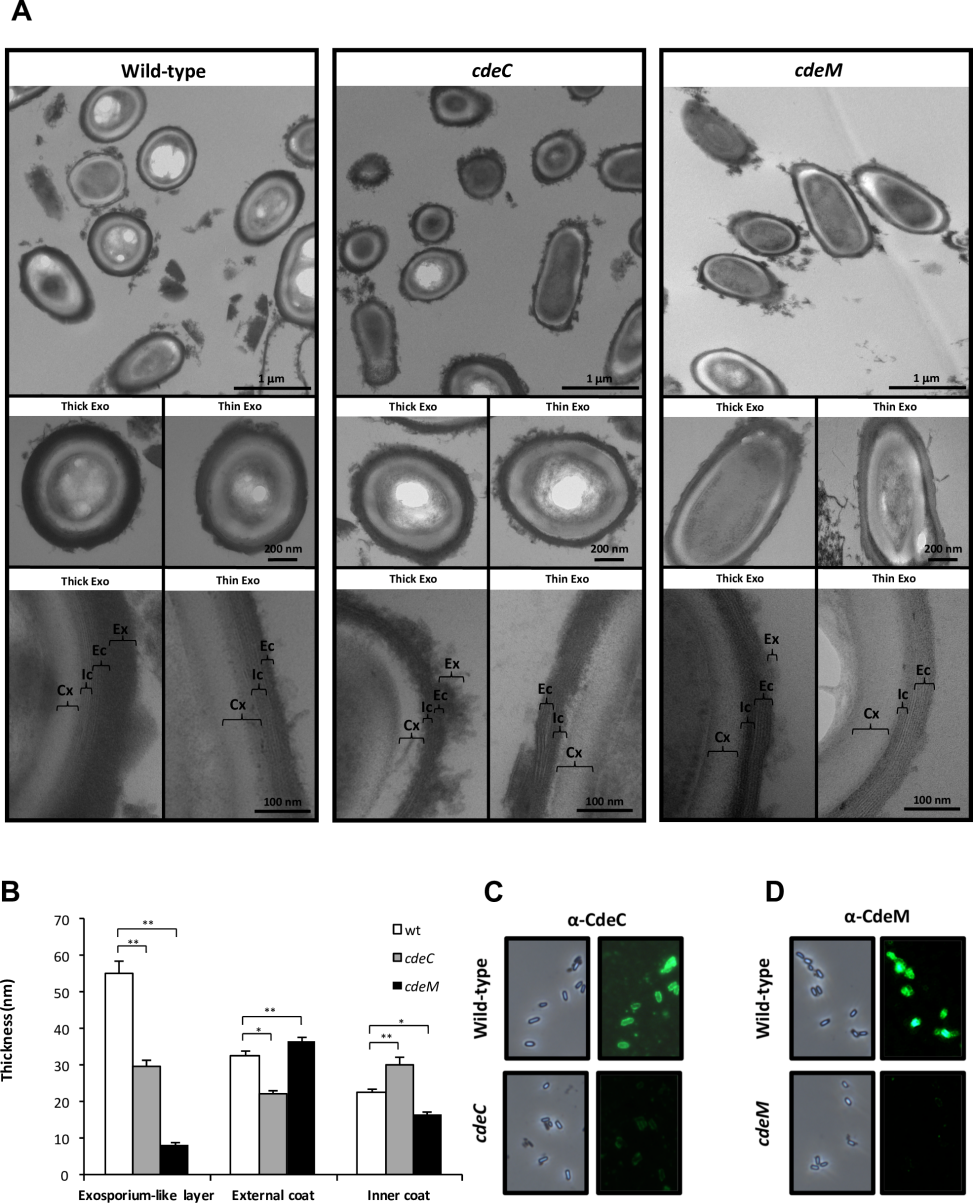
Transmission electron micrographs of *C*. *difficile cdeM* and *cdeC* spores. **(A)** Thin sections of *C*. *difficile* wild-type, *cdeC* and *cdeM* spores were analyzed by transmission electron microscopy as described in the Method section. Representative micrographs of several *C*. *difficile* wild-type, *cdeC* and *cdeM* spores are shown in the upper panel. Selected individual spores of wild-type with thick and thin exosporium layer are shown. The middle panel shows representative individual spores of the mutant strains *cdeC* and *cdeM*. The lower panel shows a magnified view of the thin section of wild-type, *cdeC* and *cdeM* spores with thick and thin exosporium layers. Ex, exosporium; Ic, inner coat; Ec, external coat; Cx, cortex. **(B)** The thickness of the exosporium and outer and inner coat layers of *C*. *difficile* wild-type (white bars), *cdeC* (gray bars) and *cdeM* (black bars) strains were analyzed by transmission electron microscopy of at least 10 individual spores with an apparent thick-exosporium morphotype. Error bars denote standard errors of the means. Asterisks (*) denote statistical difference at *P* < 0.05 and (**) denote statistical difference at *P* < 0.001 respect to wild-type. Scale bars are shown in each figure: the bars in the upper panels represent 1 nm, middle panel 100 nm and the bars in the lower panels represent 200 nm. **(C)** The surface accessibility of CdeC on *C*. *difficile* 630*erm* wild-type and *cdeC* mutant spores was analyzed by immunofluorescence with rat anti-CdeC serum as described in Methods section. **(D)** The surface accessibility of CdeM on *C*. *difficile* 630*erm* wild-type and *cdeM* mutant spores was analyzed by immunofluorescence with rabbit anti-CdeM spores as described in Methods section.

Unlike the exosporium layer of most epidemic strains, 630*erm* spores have an exosporium layer that does not exhibit bumps and the typical hair-like extensions [19, 20], and also have higher levels of CdeC in the spore surface layers than R20291 spores [23]. Given these differences, we hypothesized that CdeC would have a greater impact in exosporium and spore coat assembly than previously observed in epidemic R20291 strain [13]. Insertional inactivation of *cdeC* lead to the formation of *cdeC* spores with an outermost exosporium layer (i.e., 29.6 nm) that was 50% thinner than wild-type spores (i.e., 55 nm) (Fig 2B). We observed that inactivation of *cdeC* in 630*erm* spores affected the thickness of the spore coats (Fig 2B) to a greater extent than in our previous observations in previous observation in *C*. *difficile* R20291 epidemic strain [13]. A significant decrease of 32% (wild-type 32.8 nm and *cdeC* 22.1 nm) in the thickness of the external spore coat was evidenced in *cdeC* spores compared to wild-type spores, while an increase of 35% in the thickness of the inner spore coat was observed in *cdeC* spores compared to wild-type spores (i.e., wild-type, 22.5 nm; *cdeC*, 30.6 nm) (Fig 2B). Despite these differences, the overall thickness of the spore coat (i.e., inner coat plus outer coat) remained similar between wild-type (i.e., 55.3 nm) and *cdeC* (i.e., 52.7 nm) spores (Fig 2B). Collectively, these observations indicate that: i) CdeC affects the exosporium assembly and the thickness of the inner and external spore coat of 630*erm* spores; ii) the impact of insertional inactivation of *cdeC* in the thickness of the inner and external spore coat is greater in 630*erm* spores than in epidemic R20291 spores [13].

To explore the impact of insertional inactivation of *cdeM* in the assembly of the exosporium layer of *C*. *difficile* spores, *cdeM* spores were also analyzed by transmission electron microscopy. Strikingly, analysis of more than 50 individual *cdeM* spores revealed that inactivation of *cdeM* yielded spores with almost complete absence of the exosporium layer (Fig 2A). Upon comparison of the thickness of the exosporium layer of wild-type and *cdeM* spores (Fig 2A), we evidenced a striking decrease of 85% in the thickness of the exosporium layer of *cdeM* spores (i.e., 8.1 nm) compared to that of wild-type spores (i.e., 55 nm) (Fig 2B). In contrast to the effect of inactivation of *cdeC* on the spore coat, inactivation of *cdeM* led to a slight but significant increase in the thickness of the external spore coat layer, from 32.8 nm (i.e., wild-type spores) to 36.4 nm (i.e., *cdeM* spores) (Fig 2B). Conversely, a significant decrease in the thickness of the inner spore coat from 22.5 nm (i.e., wild-type spores) to 16.5 nm (i.e., *cdeM* spores) was observed (Fig 2B). Despite these differences, the overall thickness of the spore coat varied slightly from 55.3 nm in wild-type spores to 52.9 nm in *cdeM* spores. Collectively, these observations clearly indicate that CdeM is essential for the morphogenesis of the exosporium layer and, affects to some degree the assembly of the spore coat layer of 630*erm* spores.

The morphological defects observed as described above suggest that CdeC and CdeM are surface proteins. Indeed, previous work has demonstrated that CdeC and CdeM are located mainly in the exosporium layer [23]. To evaluate whether CdeC is surface-located, immunofluorescence of wild-type and *cdeC* spores; significant immunofluorescence signal was detectable in wild-type spores, while no detectable fluorescence signal was evidenced in *cdeC* mutant spore (Fig 2C). Similarly, immunofluorescence assay with anti-CdeM detected immunofluorescence signal in wild-type but not in *cdeM* spores (Fig 2D). These results indicate that both cysteine-rich proteins are accecible to antibodies.

### Effect of CdeC and CdeM in the abundance of the major protein species in the outer layers of *C*. *difficile* 630*erm* spores

**Fig 3:**
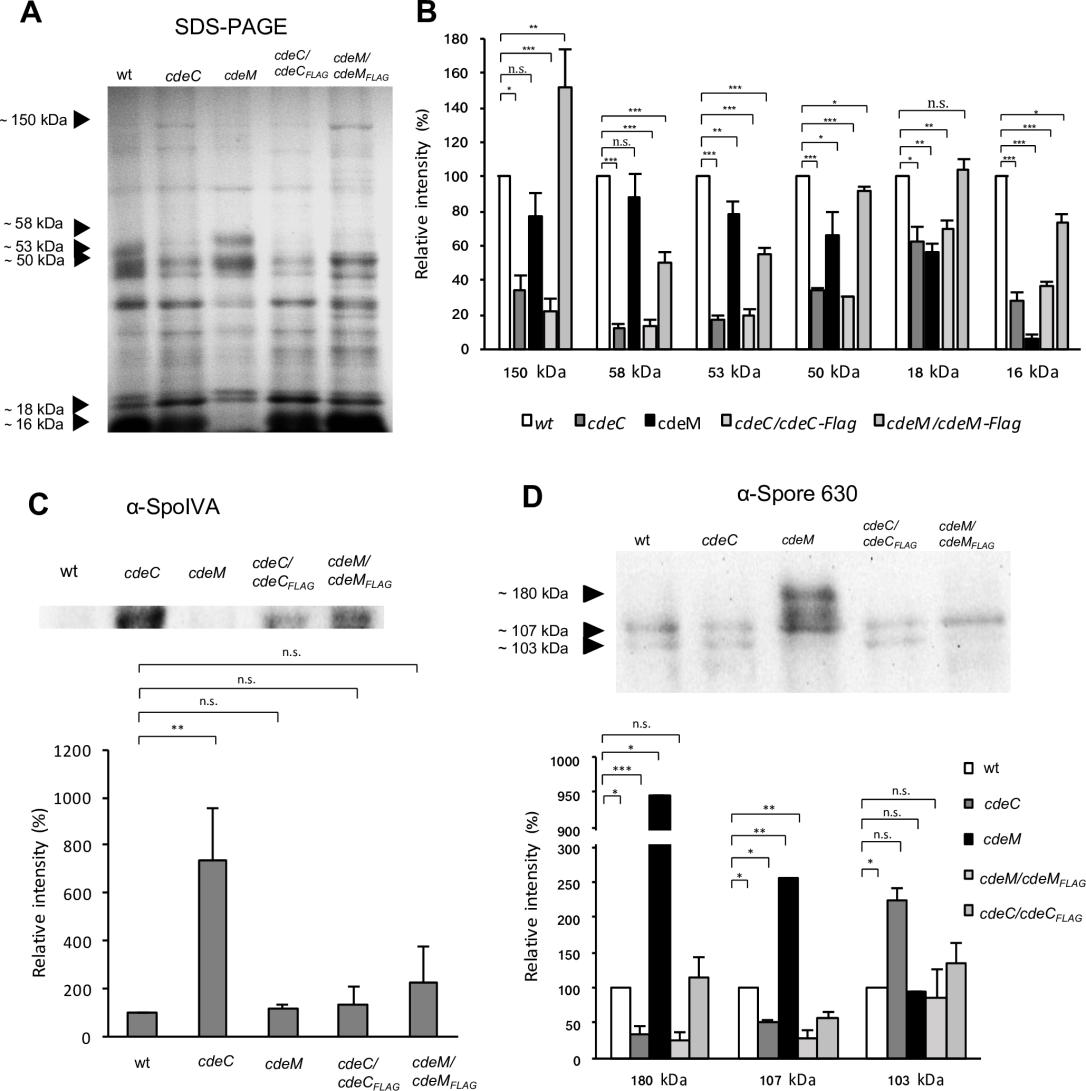
Protein profile and immunoreactive bands of *C*. *difficile* spores in the absence of CdeC and CdeM. **(A)** Coat and exosporium extracts of 4x10^7^ spores of each strain were electrophoresed and stained with Coomassie brilliant blue. Black arrows highlight the major protein bands. **(B)**, Densitometry analysis of the relative major protein bands in an SDS-PAGE gel. **(C)** Western blot of spore coat/exosporium fractions of wild-type, *cdeC* and *cdeM* spores blotted with rabbit anti-SpoIVA. Densitometric analysis of SpoIVA-immunoreactive bands were done with Image-J. **(D)**, Western blot analysis of spore coat/exosporium fractions of wild-type, *cdeC* and *cdeM* spores blotted with goat antiserum raised against *C*. *difficile* 630*erm* spore. Black arrows highlight the immunoreactive bands. Densitometry analysis of the major imunoreactive bands was determined with ImageJ, and the results are expressed as relative to those determined in wild-type spores. The SDS-PAGE and Western blots are a representative experiment. Data of densitometric analysis represent the mean of three representative experiments and error bars are standard error of the mean. Asterisks denote statistical difference at (*) *P* < 0.01, (**) *P* < 0.05 and (***) *P* < 0.001 respect to wild-type.

The fact that *cdeC* and *cdeM* spores had defective exosporium layers suggested that the protein profile of *cdeC* and *cdeM* spores might differ from that of wild-type spores. Reasoning that the protein profile would differ due to the mutations, we standardized the amounts of spores loaded by optical density, ensuring that the same number of spores were loaded in each lane. Our first observation from the SDS-PAGE analysis of the Laemmli buffer-extracted spore coat and exosporium proteins from wild-type spores was that the protein profile of 630*erm* spores differed from the previously reported one from R20291 strain [13]. Analysis of the spore coat and exosporium extracts of *cdeC* spores revealed the appearance of 6 major protein species of molecular weights estimated in 150-, 58-, 53-, 50-, 18- and 16-kDa, levels of which decreased to 34, 12, 16, 34, 63 and 28% relative to wild-type levels (Fig 3A and 3B). Strikingly, complementation of *cdeC* mutation, albeit had no effect on the levels of the 18- and 16-kDa protein species, and increased the levels of 150-, 50-kDa proteins but not to wild-type levels (Fig 3B). A similar protein profile was observed in Laemmli-extracts of the spore coat and exosporium (remnants) extracts of *cdeM* spores; the levels of the protein species of 150-, 58-, 53-, 50-, 18- and 16-kDa were decreased to 78, 88, 77, 66, 56 and 6% relative to levels in wild-type spores (Fig 3A). Complementation of the *cdeM* mutation increased the levels of most of the dominant protein species to levels near or higher than those in wild-type spores (Fig 3B). These results indicate that the absence of both cysteine rich proteins, CdeC and CdeM, affect the relative abundance of the major protein species in the spore coat and exosporium extracts.

### Effect of CdeC and CdeM on the presence of immunodominant proteins of the exosporium layer of *C*. *difficile* 630*erm* spores

Previous work, using an anti-630*erm* spore goat antiserum [13, 34], demonstrated that the immunodominant proteins are located in both, the spore coat and exosporium layer [13, 34]. Therefore, since inactivation of *cdeC* and *cdeM* affected the assembly of the exosporium layer of 630*erm* spores, we evaluated how their inactivation affects the presence of immunodominant proteins in the spore coat and exosporium extracts analyzed by western blots with anti-spore goat serum. Several loading controls of *C*. *difficile* spores have been applied recently to normalize immunoreactive intensities. Given the defects observed in the spore coat and exosporium in *cdeC* and *cdeM* spores, we first sought to evaluate whether mutations in *cdeC* and *cdeM* would affect the abundance of a loading control protein, SpoIVA, which has been used as a loading control in several studies [35, 36]. Notably, inactivation of *cdeC* caused a ~7-fold increase on the levels of SpoIVA, complementation of *cdeC* with wild-type *cdeC* restored SpoIVA levels to near wild-type level (Fig 3C). By contrast, inactivation of *cdeM* had no effect on SpoIVA levels, and complementation of *cdeM* with wild-type *cdeM* did not affect SpoIVA levels (Fig 3C). Therefore, to analyze the relative amounts of immunoreactive proteins we loaded similar amounts of spores based on optical density measurements. Analysis of the spore coat/exosporium extracts of *cdeC* spores revealed that the levels of the 180- and 107-kDa immunoreactive protein species significantly decreased 35 and 50% relative to that of wild-type spores, respectively (Fig 3D). Levels of the 103-kDa immunoreactive protein species increased by ~2-fold relative to wild-type spores (Fig 3D). Complementation of *cdeC* with wild-type *cdeC* had no effect on the levels of the immunoreactive proteins of 180- and 107-kDa; however, the levels of the 103-kDa immunoreactive protein species were restored to wild-type levels (Fig 3D). Analysis of the spore coat/exosporium extracts of *cdeM* spores revealed that the levels of the 180- and 107-kDa, but not 103-kDa, immunoreactive protein species significantly increased by 9- and 2.5-fold relative to wild-type levels (Fig 3D). Complementation of *cdeM* lead to spores with wild-type levels of all three immunoreactive protein species (Fig 3D). Collectively, these results indicate that: i) CdeC is required for the normal levels of immunoreactive protein species of the outer layers of *C*. *difficile* spores; ii) absence of CdeM leads to spores with increased levels of immunoreactive proteins.

### Absence of CdeC, but not CdeM, affects *C*. *difficile* spore coat permeability to lysozyme

**Fig 4:**
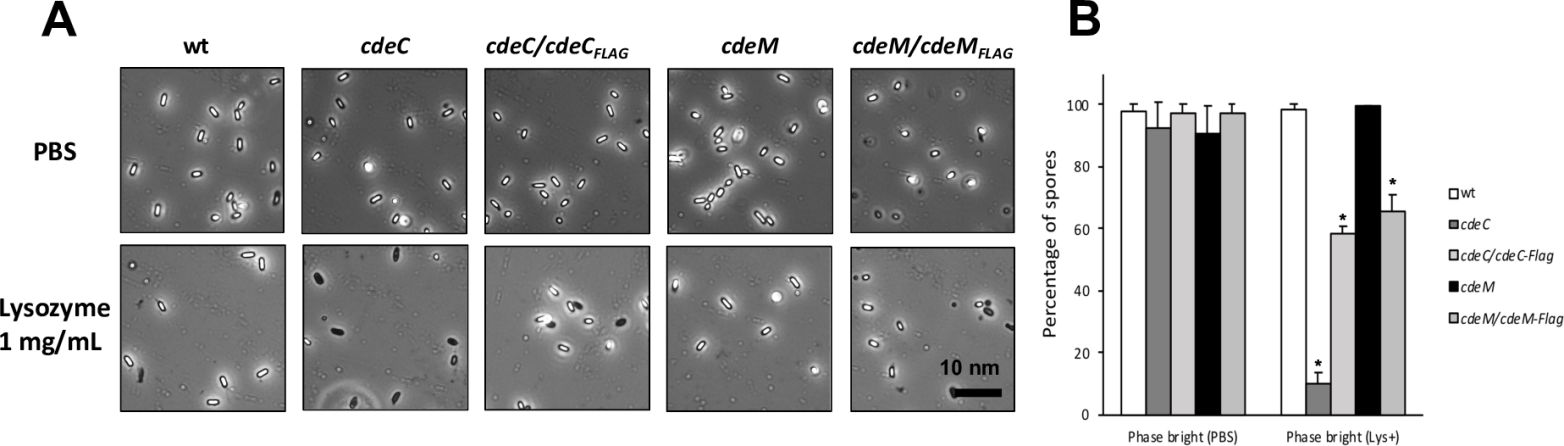
*C*. *difficile cdeC* but not *cdeM* spores are susceptible to lysozyme. **(A)** Representative phase-contrast micrographs of *C*. *difficile* wild-type, *cdeC*, *cdeM* and complemented spores treated for 5 h at 37°C with PBS (Unt) lysozyme-PBS [Lys(1 mg/mL)]. Scale bar, 10 μm. **(B)** Effect of lysozyme on the percentage of spores in bright and dark phase of wild-type, *cdeC*, *cdeC/cdeC*_FLAG_ *cdeM* and *cdeM/cdeM*_FLAG_ spores that remain dormant (white bars),. Data represent the average of three independent experiments. The bars in the panels represent 10 nm. Asterisks (*) denote statistical difference at *P* < 0.05 respect to wild-type. n.s., denotes no significance.

The spore coat of *C*. *difficile* spores acts as an impermeable barrier to enzymes with molecular masses higher than 14 kDa, such as lysozyme, proteinase K and trypsin [14]. The impact of insertional inactivation of *cdeC* and *cdeM* in the protein profile of spore coat/exosporium extracts raised the question of whether absence of CdeC and/or CdeM would impact the permeability of the spore coat to lysozyme triggered-germination. Hence, to answer this question, we explored a lysozyme permeability assay of *cdeC* and *cdeM* mutant spores. After treatment of wild-type spores with 1 mg/mL of lysozyme for 5 h at 37°C, only a small fraction of spores (1%) changed to phase dark (Fig 4A and 4B). Contrastingly, under similar treatment conditions, ~90% of *cdeC* spores changed to phase dark (Fig 4A and 4B). However, less than 1% of *cdeM* spores changed to phase dark upon lysozyme treatment (Fig 4A and 4B). *cdeC* complementation partially restored the resistance of the spore coat to lysozyme, where only 34% of the spores became phase dark (Fig 4A and 4B). Despite the negligible effect of a *cdeM* mutation in lysozyme resistance, complementation of *cdeM* strain with wild-type *cdeM* caused 38% of the spores to become phase dark after lysozyme incubation (Fig 4B). Altogether, these results indicate that, despite the impact of both cysteine-rich proteins (i.e., CdeC and CdeM) on the spore coat and exosporium proteins, only the absence CdeC increases the permeability barrier of the spore coat to lysozyme, which is consistent with those results previously reported for a insertional inactivation of *cdeC* in epidemic R20291 spores [13].

### CdeC, but not CdeM, is required for ethanol-, heat- and macrophage-resistance of *C*. *difficile* spores

**Fig 5:**
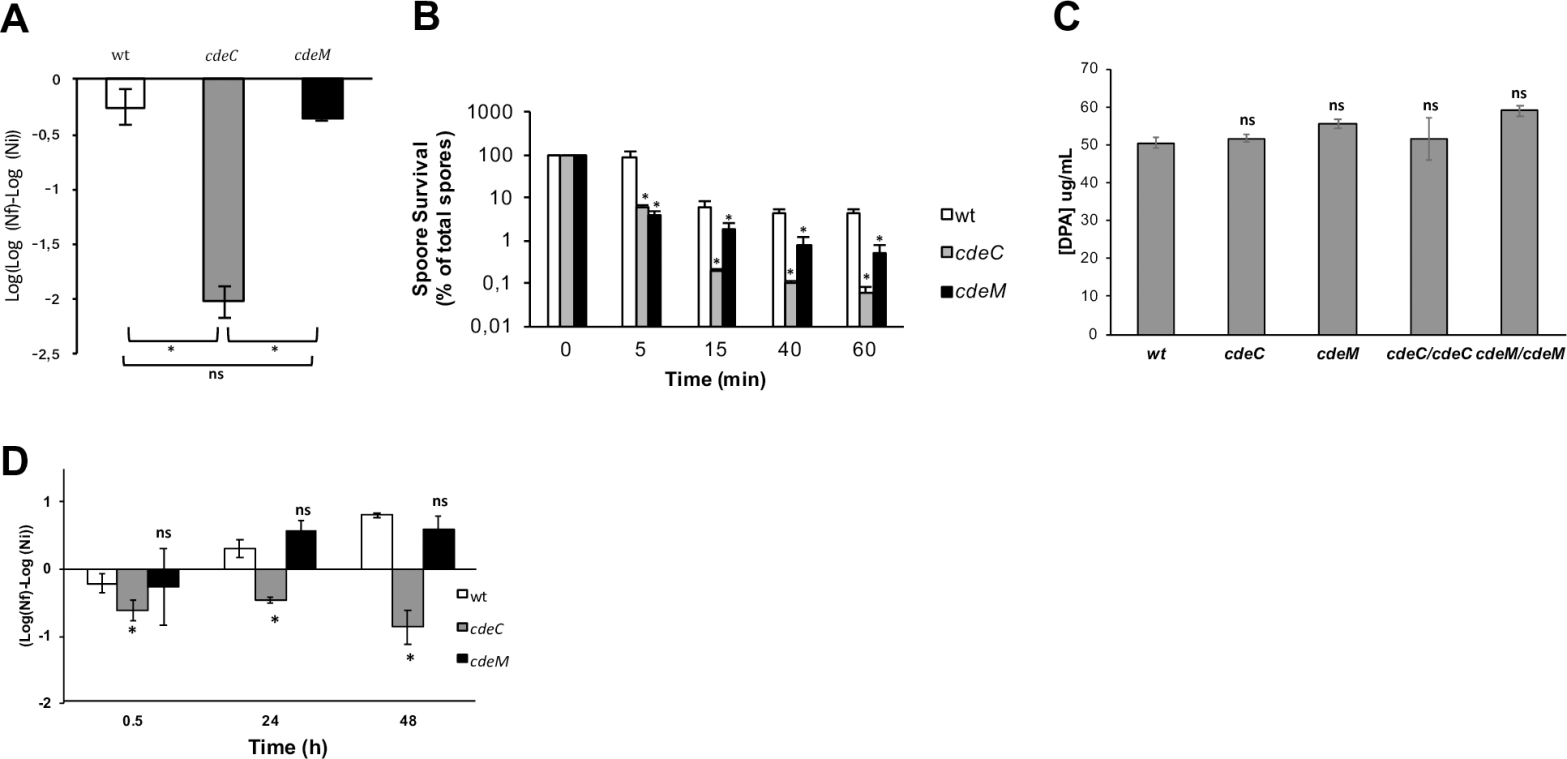
Absence of CdeC and CdeM renders *C*. *difficile* spores susceptible to ethanol, heat and macrophages. **(A)** Ethanol 50% resistance of wild-type (white bar), *cdeC* (gray bar) and *cdeM* (black bar) spores. **(B)** Heat resistance of *C*. *difficile* wild-type (gray white), *cdeC* (gray bars), and *cdeM* (black bars) spores was measured by heat treating aliquots at 75°C for various times, and survivors were enumerated as described in the Material and Method section. **(C)** Equal amounts of spores derived from *C*. *difficile* strains 630*erm* (wt), *cdeC*, *cdeM*, *cdeC/cdeC* and *cdeM/cdeM* were boiled 60 min, and the amount of DPA was quantified based on Tb^3+^. The data shown represent the average results from three independent experiments, and the error bars represent standard error from the means. n.s., indicates no significant difference relative to wild-type. **(D)** Resistance of Raw 264.7 macrophages was determined by infecting at a MOI of 10 with *C*. *difficile* 630*erm* wild-type, *cdeC* and *cdeM* after 0,5, 24 and 48 of incubation at 37°C. Asterisks (*) denote statistical difference at *P* < 0.01 respect to wild-type.

The previous work in spores of the epidemic strain R20291 demonstrated that inactivation of *cdeC* led to spores with an increased sensitivity to ethanol and heat resistance [13]. First, we evaluated whether absence of CdeC and/or CdeM affected ethanol resistance of *C*. *difficile* 630*erm* spores. Hence, when wild-type spores were treated with ethanol for 1 h at 37°C, spore viability decreased by 0.2 log reduction (Fig 5A). When *cdeC* spores were treated with ethanol under similar conditions, a significant decrease of 2 log cycles was observed (Fig 5A). By contrast, no significant difference in loss of spore viability was observed between wild-type and *cdeM* spores after ethanol-treatment (Fig 5A). These results indicate that CdeC increases ethanol-killing, presumably via an increase in the permeability of the spore inner membrane.

To gain more insight of the effects of CdeC and CdeM on resistance of *C*. *difficile* spores, heat resistance of wild-type, *cdeC* and *cdeM* spores at 75°C was assessed. Heat treatment (75°C) of wild-type spores progressively decreased spore viability (Fig 5B); after 60 min of treatment, only 4.5% of wild-type spores remained viable (Fig 5B). Upon heat treatment of *cdeC* spores, higher levels of inactivation became evident as early as 5 min after treatment (Fig 5B); after 60 min at 75°C only 0.06% of *cdeC* spores remained viable (Fig 5B). When *cdeM* spores were subjected to similar heat treatment conditions, a significantly higher extent of inactivation than wild-type was observed after 5 min at 75°C (Fig 5B). After 60 min at 75°C, only 0.5% of *cdeM* spores remained viable, amount that was 10-fold lower than wild-type spores but 10-fold higher than *cdeC* spores (Fig 5B). To address whether the decreased heat resistance of *cdeC* and *cdeM* spores was attributed to the levels of dipicolinic acid (DPA), spores of all strains were assayed for spore-core DPA content, yet no significant difference was observed in spore-core DPA content between the strains (Fig 5C). These results indicate that the absence of both exosporium morphogenetic proteins affect the resistance of *C*. *difficile* spores to heat.

*C*. *difficile* spores are resistant to phagocytic cells, and capable of surviving for more than 48 h without significant macrophage-mediated inactivation of *C*. *difficile* spores [15]. Therefore, we assessed whether the inactivation of *cdeC* and *cdeM* affected the viability of *C*. *difficile* spores during infection of Raw 264.7 macrophage-like cells. As expected, infection of Raw 264.7 cells with wild-type spores led to no significant spore-inactivation after 24 h of infection. Notably, a slight but significant increase in spore colony formation was observed after 48 h of infection (Fig 5D), suggesting that macrophage factors activated *C*. *difficile* spores to germinate in BHIS plates supplemented with taurocholate. Strikingly, while no significant inactivation of *cdeC* spores was observed after 24 h of infection of Raw 264.7 murine macrophage-like cells, ~1 log reductions in spore viability were observed after 48 of infection, respectively (Fig 5D). By contrast, no inactivation of *cdeM* spores was evidenced upon infection of Raw 264.7 macrophage-like cells after 48 of infection (Fig 5D). Collectively, these results indicate that the absence of CdeC, but not CdeM, leads to *C*. *difficile* spores susceptible to macrophage-killing.

### Effect of CdeC and CdeM in the adherence of *C*. *difficile* spore to the colonic mucosa

**Fig 6:**
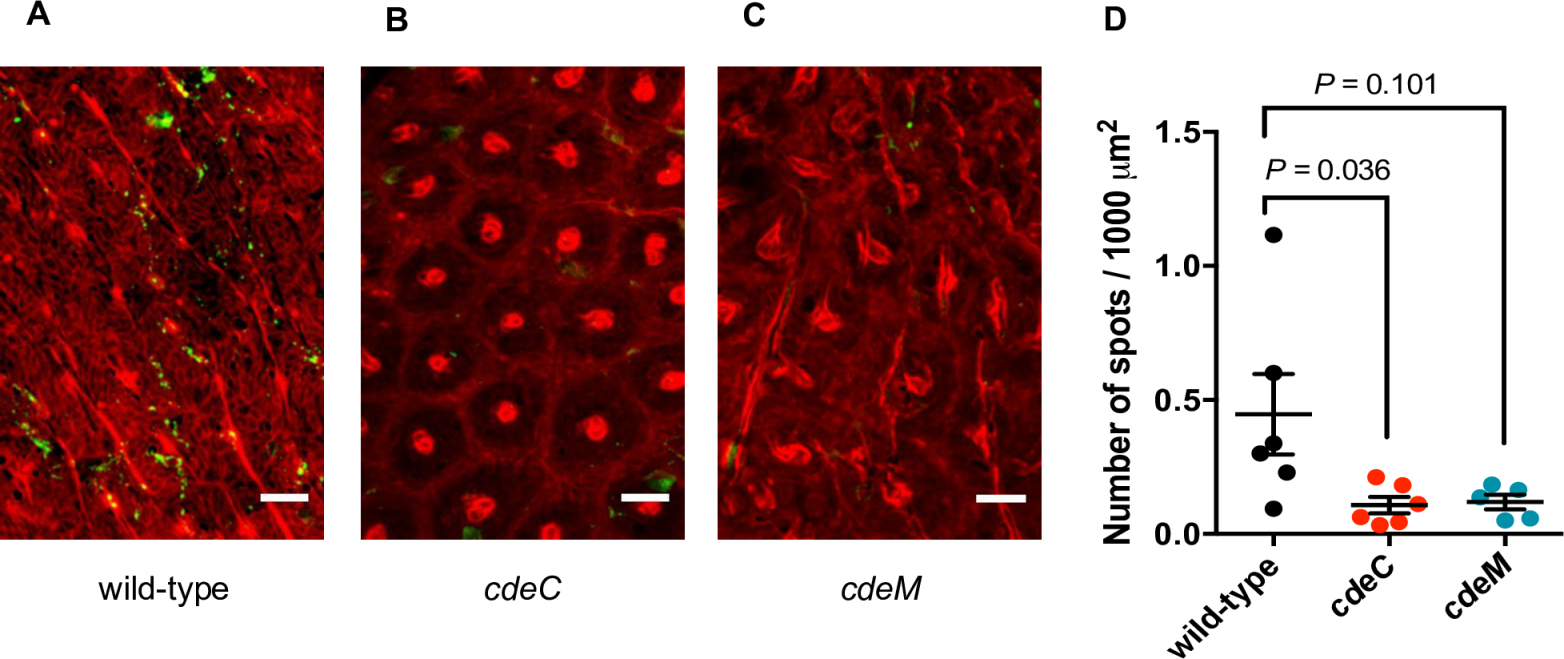
CdeC and CdeM are required for spore adherence to the colonic mucosa in a mouse model. Intestinal loops of approximately 1.5 cm of the small intestine and colon were prepared and injected with 1.3x10^8^ per cm *C*. *difficile* spores of strains wild-type (n = 6), *cdeC* (n = 6) or *cdeM* (n = 5), incubated for 5 h and processed and analyzed as described in the Material and Methods section. **(A, B, C)** Representative confocal fluorescence micrographs of colonic loops of wild-type **(A)**, *cdeC* **(B)** and *cdeM* **(C)**. **(D)** Quantification of number of spots (spores) per surface area of colonic loops as described in Material and Methods section. Kruskal-Wallis was used to detect statistical differences between the strains followed by Dunn´s multiple comparison test. Error bars indicate standard error of the mean.

Previous work demonstrated that inactivation of *cdeC* in R20291 epidemic strains lead to an increased adherence to components of the intestinal mucosa (i.e., mucin, fibronectin and adherence to intestinal epithelial Caco-2 cells) [17], suggesting that CdeC contributes to decrease the persistence of *C*. *difficile* spores in the intestinal tract. To begin answering this question, we used a colonic loop mouse model to evaluate the impact of an insertional inactivation of *cdeC* and *cdeM* in *C*. *difficile* spore adherence to healthy intestinal mucosa by confocal fluorescence microscopy (S9 Fig). In contrast to our expected results, data shown in Fig 6 demonstrates that *cdeC* mutant spores have significantly reduced adherence in comparison to wild-type spores (Kluskal Wallis test *P* = 0.036) (Fig 6A, 6B and 6D), while *cdeM* mutant spores seemed to adhere lower than wild-type to the colonic mucosa (Kluskal Wallis test *P* = 0.101) (Fig 6A, 6C and 6D). These data indicate that, in a healthy colonic mucosa, CdeC, and perhaps CdeM, contribute to reduce the adherence of *C*. *difficile* spores to the colonic mucosa, contrasting with the proposed observations from *in vitro* studies [17].

### Role of CdeC and CdeM in the initiation and recurrence of the disease

**Fig 7:**
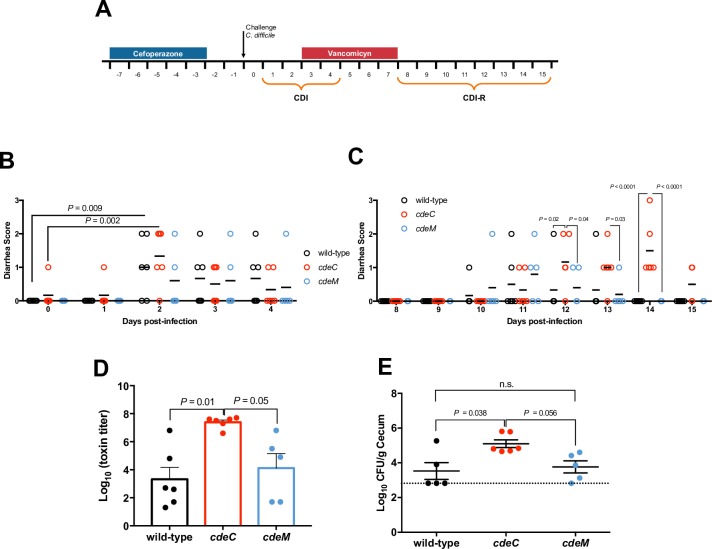
Effect of CdeC and CdeM in the initiation and recurrence of the disease in a mouse model of infection. **(A)** Overview of the experimental design for CDI-R mouse model. Cefoperazone treated C57BL/6 mice were infected with 3x10^7^ *C*. *difficile* strain 630*erm* wild-type (n = 6), *cdeC* (n = 6) or *cdeM* (n = 5). **(B)** time to diarrhea during the first episode; **(C)** animals, were treated with vancomicyn for 5 days to induce CDI-R and animals were monitored during CDI-R for time to diarrhea during recurrence; **(D)** cecum content cytotoxicity; **(E)** *C*. *difficile* spores in cecum tissue. Error bars are standard error of the mean. (Kruskal Wallis, post Dunnett test, P < 0.05); n.s, is no significance.

As mentioned, the absence of a correctly assembled exosporium layer affects spore adherence to healthy colonic mucosa. Therefore, to investigate the implication of CdeC and CdeM in an infectious context, we used a mouse model of infection and recurrent infection of *C*. *difficile*. Antibiotic-treated mice were infected with *C*. *difficile* spores of wild-type (n = 6), *cdeC* (n = 6), and *cdeM* (n = 5), and at day 3 of infection, mice were treated with vancomycin for 5 days and subsequently monitored to evaluate the recurrence of the infection (Fig 7A). Mice infected with wild-type and *cdeC* spores yielded more animals developed significantly higher diarrhea scores than those infected with *cdeM* spores (Fig 7B). Mice infected with *cdeC* spores also had higher weight lost than those infected with wild-type and *cdeM* spores (S12A Fig). Recurrence was observed after vancomycin treatment as described in Fig 7A. Diarrhea became evident at day 4 after vancomycin treatment, and 6 of 6 (100%) of the mice infected with *cdeC* developed recurrent diarrhea, whereas only 3 of 6 (50%) and 3 of 5 (60%) of the mice infected with wild-type and *cdeM* spores developed recurrent diarrhea (Fig 7C). Mice infected with *cdeC* spores also had higher diarrhea score than those infected with wild-type and *cdeM* spores (Fig 7C). The higher recurrence rate in mice infected with *cdeC* spores correlated with higher toxin titer (Fig 7D) and CFU (Fig 7E) recovered post-mortem from cecum contents. To further evaluate whether the increased colonization of *cdeC* spores could be attributed due to differences in spore germination, we evaluated whether inactivation of *cdeC* and *cdeM* affected spore germination. A reduced extent of germination in *cdeC* spores versus wild-type spores was evidenced in the presence of taurocholate after 60 min of incubation (S10A Fig). By contrast, no significant germination defect was evidenced in *cdeM* spores, which germinated similarly as wild-type spores (S10B Fig). It is also noteworthy that the colony formation efficiency of *cdeC* and *cdeM* spores in BHI agar plates with taurocholate was 25±5 and 50±5% relative to that of wild-type spores, respectively. Note that cytotoxic assay of culture supernatant on Vero cells showed no difference between strain (S11 Fig) and therefore, these parameters were not responsible for the differences observed in the *in vivo* severity and cytotoxic between strains. We also found no differences in the levels of fecal *C*. *difficile* spore loads and anti-vegetative and -spore antibodies raised during the infection (S12 Fig). Taken together, these data indicate that during the infection, insertional inactivation of *cdeC*, but not *cdeM*, leads to increased colonization and recurrence of the diarrhea after vancomycin treatment.

### Role of CdeC and CdeM in the fitness of *C*. *difficile* in a mouse model

**Fig 8:**
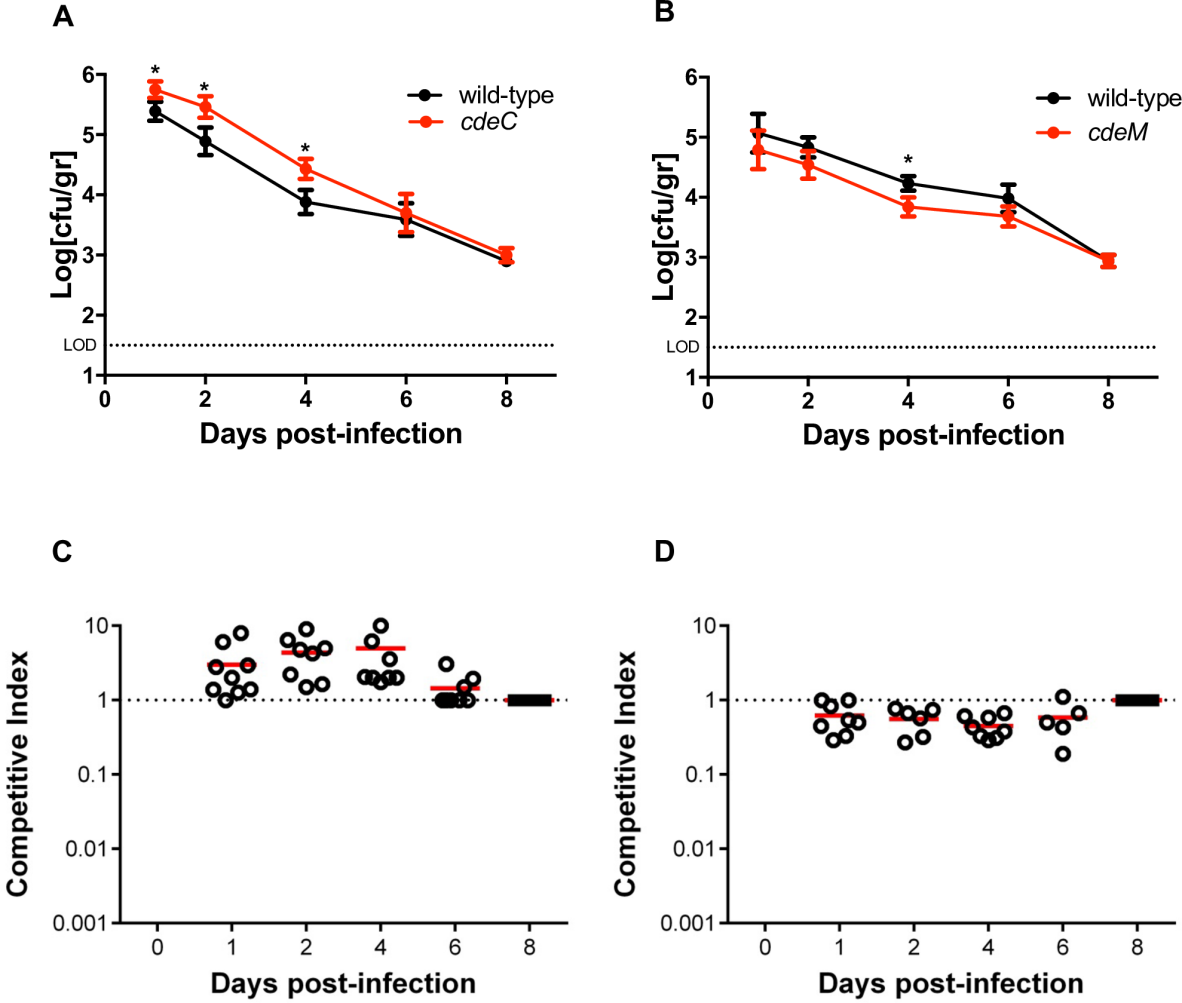
Colonization of *C*. *difficile* spores of wild-type, *cdeC* and *cdeM* strains in a murine model of infection. Mice were treated with antibiotics for 3 days, followed by intraperitoneal injection of clindamycin one day prior to infection via oral gavage with 2x10^7^ spores of a 1:1 mixture of: **(A, C)** *C*. *difficile* 630*erm* wild-type and *cdeC* mutant spores; or **(B, D)** *C*. *difficile* 630*erm* wild-type and *cdeM* mutant spores. Fecal shedding of 630*erm* wild-type and *cdeC* or *cdeM* mutants was quantified as described in the Method section. **(A, C)** Persistence of 630*erm* wild-type and *cdeC* mutant **(A)** or *cdeC* mutant **(C)** in fecal samples. **(B, D)** Competitive index (CI) course of 630*erm* wild-type and *cdeM* mutant **(B)** or wild-type and *cdeM* mutant **(D)** monitored over 8 days. Open circles indicate CI values from individual mice and the red horizontal bars indicate the geometric means. Mann-Whitney test was used to determine statistical differences between strains. Asterisks (*) indicate statistical difference with a *P*-value < 0.05. LOD, limit of detection is indicated by the dotted line.

To gain more insight on how the absence of CdeC affected *C*. *difficile* colonization, we performed a competitive assay where healthy C57BL/6 mice (n = 10 per group) were orally infected after antibiotic cocktail treatment with an equal number of viable wild-type and *cdeC* or wild-type and *cdeM* spores (1 x 10^7^ spores of each strain), and the numbers of fecal-shedded spores were monitored for 8 days after the challenge. *cdeC* spores were detected at significantly higher levels than wild-type spores at days 1, 2 and 4 post-challenge (Fig 8A and 8C). Interestingly, the persistence dynamics of *cdeM* strain differed from that of *cdeC* strain; *cdeM* spores were present at significantly lower levels than 630*erm* spores in fecal sampled only at day 4 post infection (Fig 8B and 8D). These results suggest that absence of CdeC, but not CdeM, increases the fitness of *C*. *difficile* during infection.

### Effect of inactivation of *cdeC* and *cdeM* in the presence of spore coat and exosporium proteins

To gain a better understanding on how these cysteine-rich proteins affect the assembly of the exosporium layer, we sought to evaluate the abundance of known proteins of the exosporium layer (i.e., BclA1, BclA2, BclA3, CdeA, CdeB, and CdeM) and of the spore coat (i.e., CotA and CotB) proteins [23], by using wild-type and *cdeC* mutant spores containing plasmids expressing FLAG fusion proteins (S1 Table). First, we evaluated whether the absence of CdeC and/or CdeM affected the abundance of the collagen-like BclA glycoproteins. All three BclA proteins were detectable in wild-type spores; BclA1 and BclA3 were detected forming high molecular mass complex of 110-kDa as well as a low molecular mass species of 48-kDa, while BclA2 was detectable as a 48-kDa species (S13A, S13B and S13C Fig and S14A, S14B and S14C Fig). In the absence of CdeC or CdeM, a significant reduction in the high molecular mass complex of both, BclA1 and BclA3, was evidenced (Table 1, S13A and S13C Fig, S14A and S14C Fig). By contrast, absence of CdeC leads to an increase in low molecular mass complex of all three BclA orthologues, whereas absence of CdeM leads to a decrease in the low molecular mass complex of all three BclA proteins (Table 1, S13A, S13B and S13C Fig, S14A, S14B and S14C Fig). Note that further dilution of the amount of anti-flag used provides similar results in the case of BclA1-FLAG (S15 Fig). These results demonstrate that: i) CdeC is essential for the presence of the high, but not low, molecular mass complexes of all three BclA proteins, while CdeM is essential for the presence of high and low molecular mass complexes of all three BclA proteins.

**Table 1:** Relative abundance of Flag-fusions of spore coat and exosporium proteins in *C*. *difficile* spores in the absence of CdeC and CdeM^a^.

Flag-fusion	MW (kDa)	*cdeC* mutant^b^	*cdeM* mutant^b^
**BclA1**	110	0.01 ± 0.01 (*)	0.01 ± 0.01 (*)
	48	4.08 ± 0.78 (*)	0.65 ± 0.02 (*)
**BclA2**	48	4.48 ± 0.49 (*)	0.01 ± 0.01 (*)
**BclA3**	110	0.01 ± 0.01 (*)	0.02 ± 0.003 (*)
	48	1.22 ± 0.05 (ns)	0.01 ± 0.01 (*)
**CdeA**	47	1.51 ± 0.1 (*)	1.27 ± 0.16 (*)
	19	0.01 ± 0.01 (*)	0.01 ± 0.01 (*)
**CdeB**	48	1.25 ± 0.06 (*)	0.08 ± 0.01 (*)
**CdeM**	47	1.44 ± 0.15 (*)	N.D.
**CdeC**	120	N.D.^c^	0.01 ± 0.01 (*)
	44	N.D.	0.60 ± 0.05 (*)
**CotA**	47	0.89 ± 0.03 (ns)	0.38 ± 0.003 (*)
**CotB**	47	0.15 ± 0.01 (*)	0.45 ± 0.02 (*)

^a^ Abundance of each flag fusion is expressed as fold-change relative to that of wild-type.

^b^ The data is presented as fold-change ± standard error relative to wild-type levels, where wild-type levels were set as 1. All experiments were done three independent times. Asterisk (*) denote statistical difference at *P* < 0.05; ns, denotes no significant difference.

^c^ N.D., indicates not determined.

As previously described [23], the cysteine-rich protein, CdeA, was found in the spore surface as a 19- and 47-kDa immunorreactive species (Table 1, S13A, S13B, S13C Fig and S14A, S14B and S14C Fig). Absence of CdeC or CdeM lead to a significant increase of 47-kDa CdeA species, and a significant decrease of the 19-kDa CdeA species (Table 1, S13D Fig and S14D Fig). Another exosporium protein previously identified is CdeB, which was found to be present in wild-type spores as a 48-kDa immunoreactive species as previously described [23]. Notably, while the absence of CdeC lead to a significant increase of CdeB, the abundance of CdeB in absence of CdeM lead to lower levels of CdeB compared to wild-type spores (Table 1, S13E Fig and S14E Fig). Note that further dilution of the amount of anti-flag used provides similar results in the case of CdeA-FLAG (S15 Fig). These data indicate that the levels of CdeA and CdeB are affected by CdeC and CdeM.

The aforementioned results suggest that levels of CdeC depend on the presence of CdeM or vice versa. To explore this hypothesis, levels of CdeC in *cdeM* spores relative to wild-type and levels of CdeM in *cdeC* spores relative to wild-type spores were assessed. Results evidenced that while a significant increase of CdeM was observed relative to wild-type spores (Table 1, S13F Fig). By contrast, a significant decrease in high (120-kDa) and low (44 kDa) molecular mass CdeC species was evidenced in *cdeM* spores relative to wild-type spores (Table 1, S14F Fig), indicating that spore levels of CdeC depend on CdeM.

The altered thickness of *cdeC* spores evidenced by transmission electron micrographs suggest that the absence of CdeC might affect the levels of spore coat proteins. To address this question, we evaluated the levels of two spore coat proteins (i.e., CotA and CotB) [37]. CotA and CotB were present as 47-kDa immunoreactive protein species, as reported previously in wild-type spores [23]. CotA was found at similar levels in *cdeC* spores relative to wild-type, but significantly lower levels of CotB were observed in *cdeC* spores compared to wild-type spores (Table 1, S13G and S13H Fig). Next, we addressed whether the absence of CdeM affected CotA and CotB levels. As shown in Table 1 (S14G and S14H Fig), *cdeM* spores had significantly lower levels of both CotA and CotB than wild-type spores (Table 1, S14G and S14H Fig). These results indicate that only CdeM affects CotA, but that CdeC and CdeM affect CotB.

## Discussion

*C*. *difficile* spores exhibit an outermost exosporium layer that provides the first site of interaction with the host. Recent studies on the outermost exosporium layer of *C*. *difficile* spores have uncovered the ultrastructural variability, composition and functional properties of this layer [14, 20, 23, 38–40]. Extensive studies have demonstrated that cysteine-rich proteins have been involved in the assembly of the exosporium layer of spores of members of the *B*. *cereus* group and in the outer crust layer of *B*. *subtilis* spores [18, 24–26]. In *C*. *difficile*, there are three cysteine-rich proteins identified in the exosporium layer of *C*. *difficile* spores, CdeC, CdeM and CdeA [23]. Previously, we demonstrated that CdeC is an exosporium morphogenetic protein in epidemic *C*. *difficile* strain R20291 by performing functional analysis of a *cdeC* mutant strain [13]. In this work, we have used the laboratory strain 630*erm* due to its genetic ease, to investigate how two exosporium cysteine-rich proteins, CdeC and CdeM, contribute differentially to the spore biology and pathogenesis of *C*. *difficile*: CdeC and CdeM are both required for the correct formation of the exosporium layer. Whereas *cdeC* mutant exhibit defective spore coat assembly (Fig 2A and 2B) and permeability to lysozyme (Fig 4A and 4B), increased susceptibility to ethanol, heat- and macrophage-inactivation (Fig 5A, 5B and 5C), *cdeM* spores behaved as wild-type spores. Notably, CdeC is specific to *C*. *difficile* and related Peptostreptococcaceae family members, while CdeM is specific to *C*. *difficile* (Fig 1). In a healthy colonic mucosa, spore adherence of *cdeC* and *cdeM* spores was lower than wild-type spores (Fig 6); while during infection *cdeC* mutant, but not *cdeM*, exhibited higher diarrhea score, and persistence during recurrence of infection (Fig 7). In concordance, *cdeC* mutant, but not *cdeM* mutant, exhibited increased fitness in a competitive infection mouse model. Thus, this work contributes to our understanding on the mechanisms underlying exosporium assembly, and how this impacts *C*. *difficile* spore biology and pathogenesis.

**Fig 9:**
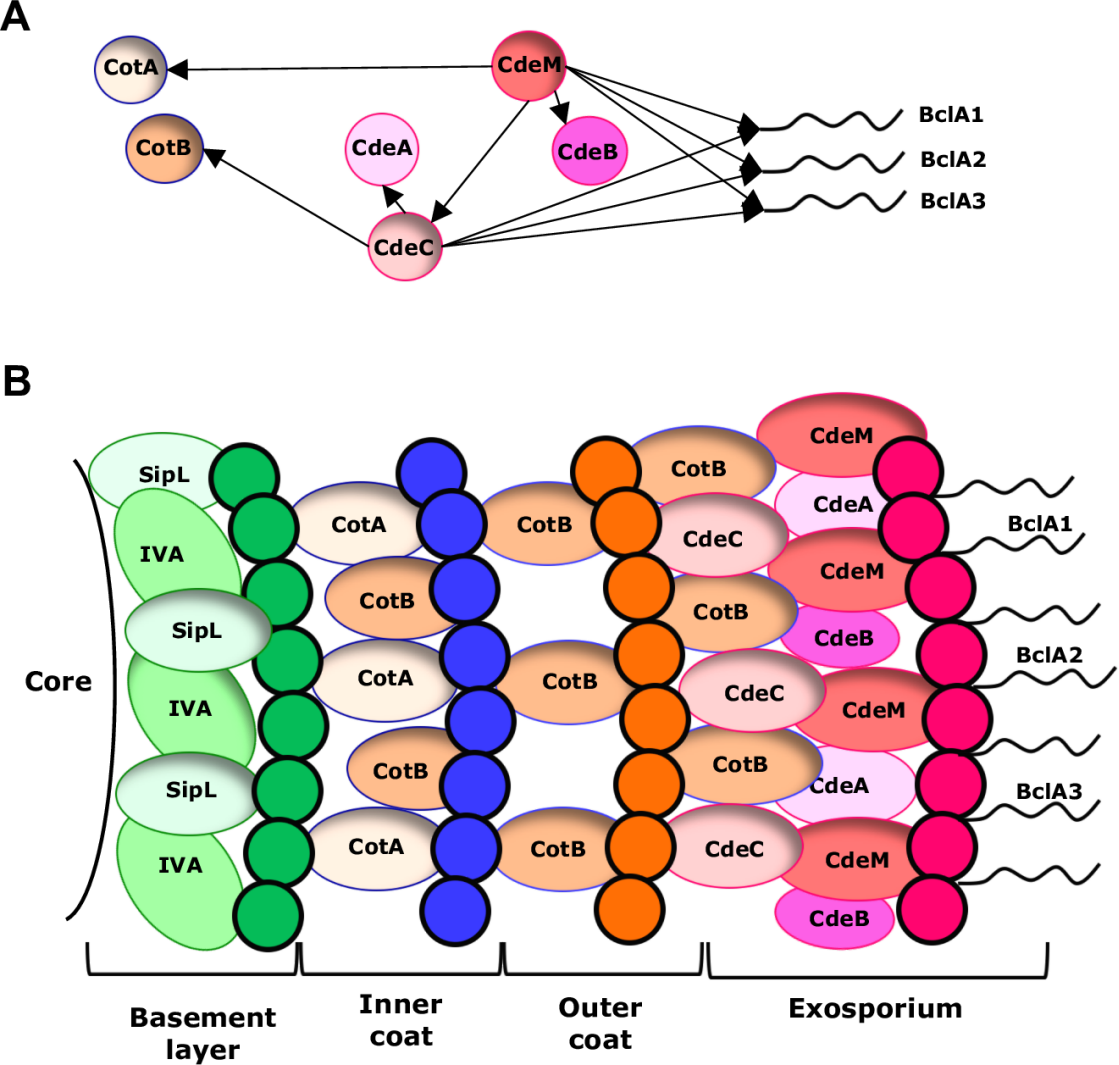
Schematic representation of proteins of the layers of the spore of *C*. *difficile* and recruitment of proteins CdeC and CdeM. **(A)** The proteins are indicated which is affected by recruitment CdeC and CdeM. **(B)** Preliminary model of the outermost layers of *C*. *difficile* spores and the putative location of spore coat (i.e., CotA and CotB), and exosporium proteins (i.e., BclA1, BclA2, BclA3, CdeA, CdeB, CdeC and CdeM) in the inner and outer spore coats and the exosporium layer of *C*. *difficile* spores.

It was surprising to observe that despite the fact that both, CdeC and CdeM, are cysteine rich proteins, they have cause differential impacts in the integrity of the exosporium layer and spore coat. *C*. *difficile* spores. Altogether, the results provided in Table 1 and S13 and S14 Figs allow the elaboration of a first interaction map and exosporium model (Fig 9A). Reasoning that we observed that the presence of CdeC was CdeM-dependent and not vice-versa (Table 1, S13F Fig and S14F Fig), CdeC-dependent proteins were defined as those with reduced levels in a *cdeC* genetic background; consequently, CdeM-dependent proteins were defined as those whose abundance were reduced in a *cdeM* but not *cdeC* genetic background. In this context, suggested CdeC-dependent proteins include CdeA, CotB and the high molecular complex BclA1 and BclA3 (Fig 9A). By contrast, CdeM-dependent proteins include CotA, CdeB, and the low molecular mass complex formed by BclA1, BclA2, BclA3 and CdeB (Fig 9A, S13 Fig and S14 Fig). It is noteworthy, that the high molecular, and to some extent, the low molecular mass complex formed by CdeC, are CdeM-dependent (Fig 9, S14F Fig and Table 1). Coupling these findings with previous localization studies [23], we propose putative locations of these proteins in the spore outer surface (Fig 9B). CotA and CotB were previously shown to be located in the spore coat layers [23], while the BclA and Cde proteins are located in the exosporium; however, the fact that the absence of CdeC affects the abundance of CotB and causes a permeable spore coat, suggests that the location of monomeric CdeC might be on the interface of the spore coat and exosporium layers, while the high molecular complex CdeC forms might be more exosporium oriented; CdeM, by contrast seems to be located uniquely on the exosporium layer. The recruitment of CotA might be related to additional unidentified proteins. Since these experiments were performed with plasmid-based complementation, we were unable to evaluate how restoring the wild-type gene into the mutant strain affected the relative abundance of FLAG-tagged proteins.

A major difference between CdeC and CdeM, was that CdeC had profound implications in the assembly and permeability of the spore coat and spore resistance. It was somewhat surprising that *cdeM* spores had an impermeable spore coat to lysozyme, while the majority of *cdeC* spores germinated in the presence of lysozyme (Fig 4). A plausible explanation could be attributed to the lower levels of the CotB, additional key spore-coat constituents or to the absence of CdeC in *cdeC* spores. It is likely that the presence of monomeric CdeC in *cdeM* spores, might sufficient to be implicated in the spore coat resistance to lysozyme, or that it might be recruiting additional constituents. However, a major question that remains unanswered, is how is CdeC, but not CdeM, implicated in spore resistance? the increased permeability of the spore coat to enzymes and of the spore inner membrane to chemicals is consistent with the elevated levels of killing of *C*. *difficile* spores to Raw 264.7 cells, where spores are subjected to low pH and a variety of stressors (i.e., release of hydrogen peroxide, lysozyme and proteases) [41], suggesting that CdeC is essential for *C*. *difficile* spores ability to survive host´s phagocytic cells. Dipicolinic acid is a known factor that contribute to heat resistance of *C*. *difficile* spores; thus, it was interesting to find that the levels of this molecule in the spore core was unaffected by the inactivation of *cdeC* and *cdeM* (Fig 5).

Another major question raised by this work is how can the absence of CdeC, but not CdeM, contribute to a decreased spore adherence to healthy intestinal mucosa, but during infection to an increased colonization, fitness and severity of the infection and recurrence? Our finding that *cdeC* spores adhere to lower levels than wild-type spores to healthy colonic mucosa in the colonic loop mouse model (Fig 6), suggests that CdeC, and/or additional exosporium proteins with reduced levels in *cdeC* spores, play a role in spore adherence to the colonic mucosa during health. By contrast, we observed an increased severity of the infection in mice infected with *cdeC* spores and increased recurrence of the infection (Fig 7C and 7E) as well as fitness (Fig 8A and 8C). A possible explanation for these contrasting observations could be attributed to the differences between a healthy and damaged colonic mucosa. For example, during infection experiments (Fig 7 and Fig 8), *C*. *difficile* toxins TcdA and TcdB cause significant remodeling of the colonic environment, including disruption of tight junctions, mucosal ulcerations and epithelial erosion [8]. These toxin-mediated epithelium damage will in turn, expose new spore-binding sites rich in extracellular matrix components to which *C*. *difficile* spores have already been shown to bind, and that include vitronectin and fibronectin [17]. Therefore, as previously shown for *cdeC* mutant spores in *C*. *difficile* R20291 genetic background, which have higher affinity against components of the intestinal mucosa such as adherence to intestinal epithelial cells, fibronectin and vitronectin [17], suggests that it is conceivable that the absence of CdeC, and/or lower additional exosporium proteins, contribute to a greater persistence of *C*. *difficile* in the host during infection, indicating that CdeC negatively contributes to *C*. *difficile* pathogenesis. In this context, the fact that 630*erm* spores have ~ 100-fold higher levels of CdeC in the spore surface than R20291 spores [23], might explain why strain R20291 is able to cause more episodes of recurrent infection than 630*erm* strain in a mouse model [42]. An increased amount of low molecular mass immunoreactive species of BclA1, BclA2 and BclA3 was observed in *cdeC* spores (Table 1, S13A, S13B and S13C Fig) that might also contribute to disease. Further studies to address how CdeC, and/or BclA proteins, contribute to interactions of *C*. *difficile* spores with components of the colonic mucosa could identify mechanism through which CdeC and/or BclA proteins modulate *C*. *difficile* spore-host interactions and may also provide insight into the mechanisms underlying the reduced adherence to healthy colonic mucosa (Fig 6), increased severity of infection and recurrence (Fig 7) and fitness during infection (Fig 8).

In summary, in identifying two cysteine rich proteins, where one is conserved (i.e, CdeM) in *C*. *difficile* and the other (i.e., CdeC) conserved in other Peptostreptococcaceae family members, our study provides insight into the mechanism of assembly of the exosporium layer of *C*. *difficile* spores and in the implications of these proteins during *C*. *difficile* infection. While many unanswered questions remain, the correct assembly of the exosporium layer is subjected to CdeC and CdeM, where CdeC seems to have a pleiotropic role in the assembly of *C*. *difficile* spores, contributing to spore resistance and persistence as well. By contrast, given that CdeM is unique to *C*. *difficile*, it can be considered as a potential target for spore-targeted therapies given the limited conservation of CdeM in other spore-forming organisms.

## Material and methods

### Ethics statement

All experiments using mice were conducted in agreement with the ethical standards and according to the local animal protection law. All experimental protocols were conducted in strict accordance with, and under the formal approval of the Institutional Animal Ethics Committee of the Universidad Andrés Bello (Protocol number 020/2010 and 026/2018) in strict accordance to the Chilean national Law 20.380. Once experiments finalized, animals were sacrificed by euthanasia by 4 times the anesthetic doses of ketamine/xylazine combinations were administered intraperitoneally. The name of the Universidad Andrés Bello Institutional Animal Care and Use Committee is: “Comité de Bioética de la Vicerrectoría de Investigación y Doctorados”. The "Comité de Bioética" provided ethical approved in the Acta # 014/2015.

### Bioinformatic analysis

Genome assemblies for selected strains (shown in Fig 1) were obtained via ftp from NCBI Assembly which included genomes of 336 Peptostreptococcaceae (taxid:186804), 214 Lachnospiraceae (taxid:186803) and 338 Clostridiaceae (taxid:31979). Many of these genomes were incomplete and were not annotated completely, therefore they were reannotated using Prodigal v2 2.6.3 [43]. A database of predicted proteins was created and searched locally using makeblastdb tool from the BLAST+ 2.3.0 package [44] using the *C*. *difficile* 630*erm* CdeC and CdeM proteins as queries (UniProt id: Q18AS2 and Q186D6, respectively). Since CdeC and CdeM have no protein motives, in order to reduce the number of false positive hits, we used blastp instead of delta- and psi-blast. Matching proteins with a threshold < 50 bits [45]. Multiple sequence alignment was performed using localpair FLAG of MAFFT v7.294b [46]. The inference of phylogenetic trees was calculated using distance-based UPGMA model of Segotron [47]. The logo was created using Seq2Logo V2.0 [48] with a minimum stack width of 0.1 and probability weighted Kullback-Leibler Logo.

### Bacterial and cell culture conditions

The *C*. *difficile* and *Escherichia coli* strains used in this study are described in S1 Table. *C*. *difficile* was routinely grown under anaerobic conditions using a gas mixture containing 90% N_2_, 5% CO_2_, 5% H_2_. Culture medium was 3.7% Brain Heart Infusion supplemented with 0.5% yeast extract and 1% cysteine (BHIS) broth or on 1.5% agar BHIS plates. Caco-2 cells were grown in Dulbecco's modified Eagle's minimal essential medium (DMEM) (Hyclone, U.S.A). All media were supplemented with 10% (v/v) fetal-calf serum (Hyclone, U.S.A.), penicillin (100 IU mL^-1^) and streptomycin (100 μg mL^-1^).

### Construction of both *C*. *difficile cdeC* and *cdeM* mutants and complemented strains

Two derivatives of *C*. *difficile* strain 630*erm* with an intron inserted in *cdeC or cdeM* genes, respectively, were constructed as follows. To target the *L1*.*ltrB* intron to each gene *cdeC* or *cdeM*, we used plasmid pDP306 and pDP370 (S1 Table). Three short sequence elements from the intron RNA involved in base pairing with the DNA target sites were modified by PCR, using *cdeC*-specific primers P68, P69, P70 and universal primer IBS described elsewhere [13]; and *cdeM* specific primers P85 (5'-AAAAAAGCTTATAATTATCCTTACAGTTCGAACCTGTGCGCCCAGATAGGGTG-3'), P86 (5'- CAGATTGTACAAATGTGGTGATAACAGATAAGTCGAACCTCTTAACTTACCTTTCTTTGT-3') and P87 (5'-TGAACGCAAGTTTCTAATTTCGGTTAACTGTCGATAGAGGAAAGTGTCT-3'). The clostron plasmids pDP306 or pDP370 were transformed into *E*. *coli* HB101 (pRK24) and subsequently transferred through conjugation to *C*. *difficile* strain 630*erm*. Thiamphenicol resistant clones were selected and re-grown on BHIS plates containing thiamphenicol and FeSO_4_ to induce expression of the Targetron system. Erythromycin-resistant clones were selected and then isolation streaked on BHIS plates supplemented with erythromycin (5 μg/mL). Positive clones were screened by colony PCR for a 2.8-kb insertion in *cdeC* with pair primer P62 (5'-GAATTTACTTAGCCACCGGTGTTTCGGG-3'), P63 (5'-TTTCTTCCTACTATATCTCCTAATGGGTCTAAATCG-3'), and *cdeM* with pair primer P83 (5'-GACCATATGGAAAATAAAAAATGTTATTCAGAAGATTGGTATGAAAG-3'), P84 (5'-GACGGATCCGATTTCCATTTCTTCTAGCTTCACATTCC-3'), (S8 Fig). Three independent clones were phenotypically characterized.

To evaluate whether the observed *cdeC* and *cdeM* phenotypes were attributed to inactivation of *cdeC* and *cdeM*, these strains were complemented with *cdeC*- and *cdeM*-FLAG fusions using plasmids pDP345 and pDP360 (S1 Table). Briefly, *C*. *difficile* 630*erm cdeC* and *cdeM* mutants were complemented by conjugating with *E*. *coli* HB101 containing plasmids pDP345, pDP360, pPCR3 and pPCR4 respectively (S1 Table). Trans conjugants were selected in BHIS agar plates containing erythromycin and thiamphenicol.

### Spore purification

Spore suspensions were prepared by plating a 1:100 dilution of an overnight culture onto a 70:30 medium (63 g Bacto peptone (BD Difco), 3.5 g proteose peptone (BD Difco), 0.7 g ammonium sulfate (NH_4_)_2_SO_4_, 1.06 g Tris base, 11.1 g brain heart infusion extract (BD Difco) and 1.5 g yeast extract (BD Difco) for 1L) and incubating it for 7 days at 37°C under anaerobic conditions. After incubation, plates were removed from the chamber and the surface was scraped up with ice-cold sterile water. Next, the spores were washed five times gently with ice-cold sterile water in micro centrifuge at 14,000 rpm for 5 min. Spores were loaded onto a 50% Nycodenz solution, centrifuged (14,000 rpm, 40 min). After centrifugation, the spores pellet was washed five times (14,000 rpm, 5 min) with ice-cold sterile water to remove Nycodenz remnants. The spores were counted in Neubauer chamber and volume adjust at 5x10^9^ spores per mL.

### Transmission electron microscopy

To analyze the ultrastructure of spores of *C*. *difficile* 630*ermB* wild-type, *cdeC* and *cdeM* mutant spores (~2x10^8^) were fixed with 3% glutaraldehyde and 0.1 M cacodylate buffer (pH 7.2) overnight at 4°C, and stained for 30 min with 1% tannic acid. Samples were further processed and embedded in spurs resin as previously described [38]. Thin sections obtained with a microtome were placed on glow discharge carbon-coated grids and double-lead stained with 2% uranyl acetate and lead citrate. Grids were analyzed with a Phillips Tecnai 12 Bio Twin at the Electron Microscopy facility of the Pontificia Universidad Católica de Chile.

### Immunofluorescence of *C*. *difficile* spores

*C*. *difficile* wild-type, *cdeC* and *cdeM* mutant spores were fixed with 3% paraformaldehyde (pH 7.4) for 20 min in poly-L-lysine-coated glass cover slides. Fixed spores were rinsed three times with PBS and blocked with 1% bovine serum albumin (BSA) for 30 min and further incubated for 2 h at room temperature with primary antibodies 1:50 of rat antiserum raised against CdeC [13] or with 1:100 of rabbit antiserum raised against CdeM (kindly provided by Dr. Adriano Henriques, Universidade Nova Lisboa). Next, covers containing fixed spores were incubated for 1 h at room temperature with 1:500 anti-rat IgG-Alexa488 conjugate (Thermo Fisher) or with 1:500 anti-rabbit IgG-Alexa488 conjugate (Thermo Fisher) in PBS-1% BSA and washed three times with PBS and once with distilled water. Dried samples (30 min at room temperature) were mounted with Dako fluorescence mounting medium (Dako North America) and sealed with nail polish. Samples were analyzed with a BX53 Olympus fluorescence microscope.

### Western blot analysis

Samples (10 μl) of coat and exosporium extracts of 5x10^7^ spores of *C*. *difficile* 630*erm* wild-type and *cdeC* or *cdeM* mutant strains were treated twice at 100°C for 5 min in SDS-PAGE loading buffer and run on SDS-PAGE gels (12% acrylamide). Proteins were transferred to a nitrocellulose membrane (Bio-Rad) and blocked for overnight at 4°C with 2% bovine serum albumin (BSA) in TBS (pH 7.4). These western blots were probed with a 1:1,000 dilution of anti-FLAG for 1 h at room temperature and then with 1:10,000 dilution of anti-mouse-horseradish peroxidase (HRP) conjugate (Promega) for 1 h at room temperature in PBS 1X with 1% BSA and 0.05% Tween20. In the western blot with goat antiserum raised against *C*. *difficile* 630*erm* spore [30] and anti-SpoIVA (kindly provided by Dr. Shen Tufts University, U.S.A.), after the transference, the nitrocellulose membrane was blocked for 1 h at room temperature with 10% milk–Tris-buffered saline (TBS) (pH 7.4). These western blots were probed with a 1:500 goat antiserum raised against spores of *C*. *difficile* 630*erm*, 1:2500 rabbit antiserum raised against SpoIVA [32] for 1 h and then with a 1:10,000 dilution of anti-goat and anti-rabbit horseradish peroxidase (HRP) conjugate (Promega) for 1 h at room temperature in PBS–1X BSA–0.1% Tween 20. In both cases, HRP activity was detected with a chemoluminescence detection system (Fotodyne Imaging system) by using Pico*Max* sensitive chemiluminescent detection system HRP substrate (RockLand Immunochemicals). Each western blot also included 1 μl of PageRuler Plus prestained Protein Ladder (Fermentas). Each western blot was repeated at least 3 independent times, and analyzed by densitometry to quantify the relative amounts of protein by ImageJ as previously described [13]. Antibodies against SpoIVA were a gift from Dr. Aimee Shen [36].

### Spore colony forming efficiency

To quantify the effect of a *cdeC* and *cdeM* mutation on spore forming efficiency, aliquots of *C*. *difficile* 630*erm* wild-type and *cdeC* and *cdeM* spores (1x10^7^ spores/mL) were plated with or without a heat activation (65°C, 20 min) onto BHIS-ST agar plates and incubated anaerobically for 36 h at 37°C. Spore viability was calculated using the following formula: [(c.f.u. mL^-1^)/(spore particles mL^-1^)] x 100 and expressed relative to wild-type strain.

### Spore resistance treatments

Ethanol resistance of *C*. *difficile* 630*ermB* wild-type, *cdeC* and *cdeM* spores was measured by resuspending 3x10^6^ spores in 30 μl of 50% ethanol in PBS 1X. Spores were incubated with ethanol for 30 min at 37°C and shaking (200 rpm). Aliquots were plated onto BHIS-ST agar plates and incubated anaerobically for 36 h at 37°C.

Heat resistance of *C*. *difficile* spores was determined as previously described [13]. Briefly, 3x10^6^ spores of strains *C*. *difficile* 630*erm* wild-type, *cdeC* and *cdeM* were resuspended in 30 μl of PBS 1X pH 7.4 and heat treated at 75°C for 60 min. Aliquots at appropriate dilutions were plated onto BHIS-ST agar plates and incubated anaerobically for 36 h at 37°C. As a control of non-heat-treated spores, an aliquot was plated onto BHIS-ST agar plate prior to the experiment and colonies counted as described above.

*C*. *difficile* spore-lysozyme resistance was measured by resuspending 3x10^6^ spores in 30 μl of PBS 1X with 1 mg/mL of lysozyme and incubated for up to 5 h at 37°C with shaking (200 rpm). Germinated spores were analyzed by phase contrast microscopy. Spore viability was measured by plating aliquots onto BHIS-ST agar plates and incubated anaerobically at 37°C for 36 h and colonies counted. In some experiments, lysozyme-treated *C*. *difficile* 630*erm* wild-type, *cdeC* and *cdeM* spores were subsequently treated with 50% ethanol for 30 min at 37°C with shaking (200 rpm) and aliquots plated onto BHIS-ST agar plates and colonies counted after 36 h of incubation under anaerobic conditions.

### DPA assay

To quantify spore-core DPA content, 200 μl of 5x10^9^ spores/ml were boiled 60 min, cooled on ice for 2 min, centrifuged at 14,000 rpm x 5 min, and 190 μl of the supernatant was mixed with 10 μl 800 μM TbCl_3_ in a 96-well plate, and DPA release was monitored with an excitation of 270 nm and emission of 545 nm in a Synergy H1 Hybrid Multi-Mode Reader (BioTek) as described [49, 50].

### Infection of Raw 264.7 macrophages

To measure the adherence of *C*. *difficile* 630*erm* wild-type *cdeC* and *cdeM* mutant spores to Raw 264.7 cells (ATCC, U.S.A.), a 96-wells plate was seeded (5x10^5^ cells per well) and incubated at 37°C in 5% CO_2_ atmosphere. Confluent Raw 264.7 monolayers were infected with 40 μl of RPMI containing *C*. *difficile* 630*erm* wild-type, *cdeC* and *cdeM* spores at an MOI of 10. After 30 min of incubation at 37°C, macrophages were washed three times with PBS 1X to rinse out unbound spores. Infected macrophages were lysed with 0.01% Triton X-100, and adhered spores were counted by plating appropriate aliquots onto BHIS-ST agar plates and incubated for 36 h anaerobically at 37°C. Colonies were counted and expressed as c.f.u. mL^-1^ for colony counts, no additional colonies appeared upon further incubation periods. Total spores were counted by lysing the infected macrophages prior to rinsing off the unbound spores and plating appropriate dilutions onto BHIS-ST agar plates and colonies counted after 36 h of incubation at 37°C under anaerobic conditions.

To evaluate *C*. *difficile* spore survival during infection of macrophages, after monolayer of Raw 264.7 cells were washed three times with PBS, macrophages were infected at an MOI of 10 as described above and unbound spores were rinsed off with three washes with PBS and macrophages were resuspended in 80 μl of RPMI with FBS 1% (to avoids macrophage replication). Viability of *C*. *difficile* spores was determined at 0.5, 24, 48 and 72 after infection by lysing infected macrophages with 0.01% Triton X-100, and serial dilutions plated onto BHIS-ST agar plates.

### Germination assay

The purified spores were heat activated for 30 min at 60°C. Next, were diluted in BHIS only or BHIS supplemented with 10 mM sodium taurocholate (Sigma-Aldrich). Heat-activates spores in BHIS only was used as control. The OD_600_ was monitored immediately (zero time) and various times for 1h at 37°C.

### Cytotoxicity of *C*. *difficile*

To determine citotoxicity of *C*. *difficile* strains an aliquot from a *C*. *difficile* was inoculated into BHIS broth and incubated for 24 h at 37°C under anaerobic conditions. Next, 1 mL of a 24-h BHIS culture was centrifuged and filtered and diluted 1:100 in Dulbecco Minimum Eagles Medium (Lonza, USA) supplemented with 10% filtered fetal bovine serum and 100 μL to each well of a 96-well plate containing Vero cells. The cells were incubated at 24 h under 5% CO_2_. The circularity of the cells was recorded (more than 50% of the cells). The cytotoxicity was measured with the following formula: Log_10_ ((percentage of rounded cells) x 100).

### Animals

6-8 weeks old C57BL/6 (male or female) were obtained from breeding colony at the Facultad de Odontología de la Universidad de Chile (Santiago, Chile) that was originally established using animals purchased from Jackson Laboratories. All mice used in the experiments were housed individually cages and were acclimated for 1 week at the Animal Infection Facility of the Microbiota-Host Interactions and Clostridia Research Group at the Universidad Andrés Bello before the experiment. Water, bedding and cages were autoclaved, and mice has a 12-hour cycle of light and darkness.

### Competitive colonization assays

The *C*. *difficile* murine model of infection was used to perform competitive index (CI) experiments. For each competitive assay, wild-type C57BL/6 mice (n=5) were challenged with 10^7^ spores via gavage in 0.2 mL PBS. Equal amounts of spores (5x10^6^) from the parental wild-type 630e*rm*, *cdeC* and *cdeM* mutant were used. Fecal samples were collected and enumerated by plating on TCCFA agar, with and without erythromycin, and incubated for 48 h. Agar supplemented with erythromycin selected for the knockout containing the *ermB* cassette. The CI number was determined using the following ratio: [(630 *cdeC* or *cdeM*/630 wild-type) output] / [(630 *cdeC* or *cdeM*/630 wild-type) input]. Statistical testing was performed using the Mann Whitney test applied to Log_10_ values of the CI ratios.

### Mouse model of recurrent infection

To induce *C*. *difficile* susceptibility in mice, prior the infection mice were administrated with a wide spectrum antibiotic, cefoperazone (0,5mg/mL) (Sigma) in drinking water for 5 days, following 2 days of normal water as has been previously described [51, 52]. Then animals were orogastrically infected with 3x10^7^ *C*. *difficile* spores strain 630*erm* (n = 6); *cdeC* (n = 6) or *cdeM* (n = 5). All procedures and mouse handling were performed aseptically in a biosafety cabinet to contain spore-mediated transmission. To evaluate recurrence of CDI, from days 3 to 9, all groups of mice were orogastrically administered 100 μl of PBS containing vancomycin (50 mg/kg; Sigma-Aldrich). During all the experiment, mice were daily monitored, and weight loss and diarrhea score and *C*. *difficile* spore shed. Sickness behaviors monitored daily, and fecal samples, and at the end of the assay, animals were sacrificed with a lethal dose of ketamine/xylazine and cecum content and colonic tissue were collected. The clinical condition of mice was monitored daily with a scoring system (CDI). The presence of diarrhea was classified according to severity as follows: (i) normal stool (score = 0); (ii) color change/consistency (score = 1); (iii) presence of wet tail or mucosa (score = 2); (iv) liquid stools (score = 3). A score higher than 0 was considered as diarrhea [52].

### Quantification of spores from feces and colon

Collected fecal samples were stored at -20°C until spore quantification. Feces were hydrated with 500 μL sterile MilliQ water ON at 4°C and then added 500 μL of absolute ethanol (Merck) and at RT incubated for 60 min. Serially diluted of sample were plated on onto selective medium supplemented with taurocholate (0.1% w/v), Cefoxitin (16 μg/mL), L-cycloserine (250 μg/mL) (TCCFA plates). The plates were incubated anaerobically at 37°C for 48 h, colonies counted, and results expressed as the Log_10_ [CFU/g of feces] [52].

Colonic tissue was collected from mice, washed three times with PBS with a syringe. The spore load in the colon was determined in two sections: proximal colonic tissue, medium colonic tissue and distal colonic tissue and cecum tissue. First proximal colonic tussue was collected in three sections (proximal, medium, distal) and the first cm of each section (from the cecum) was obtained. For cecum tissue 1 cm from the base was obtained. After, tissue was weighted, and PBS: Absolute ethanol (1:1) was added (10 μl/mg of tissue), homogenized and incubated by 1 hour. The amounts of spores were quantified plating the tissue homogenization onto TCCFA plates. The plates were incubated anaerobically at 37°C for 48 h. Finally, the colony count was expressed as the Log_10_ [CFU/gram of colon].

### Cecum content cytotoxicity assay

Vero cell cytotoxicity was performed as described previously [51]. Briefly, 96-well flat bottom microtiter plates were seeded with Vero cells at a density of 10^5^ cells/well. Mice cecum contents were suspended in PBS at a ratio of 1:10 (10 μL of PBS per mg of cecum content), vortexed and centrifuged (14,000 rpm, 5 min). Filter-sterilized supernatant was serially diluted in DMEM supplemented with 10% FBS and 1% penicillium streptomycin; 100 μL of each dilution was added to wells containing Vero cells. Plates were screened for cell rounding 16 h after incubation at 37°C. The cytotoxic titer was defined as the reciprocal of the highest dilution that produced rounding in at least 80% of Vero cells per gram of luminal samples under X200 magnification.

### Detection of *C*. *difficile* spore and vegetative cells by serum from challenged mice

Serum from infected animals were tested against or 630*erm* vegetative cells by ELISA. 1.6x10^7^ spores or 3.0 x 10^6^ vegetative cells prefixed in PFA 4% per well were incubated in 96-wells plate by 16 hrs at 4°C. Plates were washed with PBS- Tween20 0.05%, 3 times and blocked with 2% BSA by 1 hr at 37°C. After 3 washes, wells were incubated with serum dilutions 1:200, and incubated for 2 hr at 37°C. After 5 washes, secondary anti-mouse HRP antibody was added at 1:10,000 and incubated at 30°C for 1 hour and finally washed 5 times. Colorimetric reaction was initiated upon addition of 50 μL of reaction buffer (0.05 M citric acid, 0.1 M disodium hydrogen phosphate) containing 2mg/mL of o-phenlyendiamine (Sigma-Aldrich, U.S.A.) and 0.015% of H_2_O_2_ (Merck, Germany). Reaction was stopped after 20 min with 25 μL of 4.5 N of H_2_SO_4_ and absorbance was measured at 492 nm. Background reactivity was performed using IgY from eggs obtained prior immunization.

### Intestinal loop assay

Before to surgery mice were deeply anesthetized in a general way with Small Animal Anesthesia Machine for which the mice were induced in a chamber with 5% isoflurane (RWD), then the mice were maintained with 1.5% isoflurane during the surgery administered by air. Briefly, after a midline laparotomy, 1.5 cm ileal and proximal colon were ligated and injected with 3.3x10^8^ spore/cm in 0.1 mL of PBS (pH 7.2) for intestinal loops (n = 6 for wild-type and *cdeC*; n=5 for *cdeM*). The abdomen was closed with superglue, and the animals were allowed to regain consciousness. The mouse was kept for 5 h at which time the animal was euthanized, and the ligated loops were removed and washed gently in PBS and fixed in 4% paraformaldehyde 30% sucrose during 16h, washed and subjected to indirect immunofluorescence. Tissue were made permeable by incubation with 0.2% Triton X-100 in PBS 1X and blocked with 3% BSA in PBS for 3h. Tissue were made permeable by incubation with 0.2% Triton X-100 in PBS 1X and blocked with 3% BSA in PBS for 3h. The same buffer was used for subsequent incubation with antibodies. Intestine fragments were incubated with a primary polyclonal IgY anti-*C*. *difficile* spore and fluorescently labelled phalloidin (Alexa Fluor 568) for 12-16h at 4°C. Following PBS washed, samples were reacted with goat anti-chicken IgY secondary antibodies (Alexa Fluor 488) and Hoechst. For mounting was applied a drop of DAKO fluorescent mounting medium onto the tissue segment and mount cover glass over it and sandwich the tissue section. The ends of the cover glass should be fixed to the glass slide with a vinyl tape to hold the tissue sections in place.

### Confocal microscopy and imaging analysis

To acquiring images Leica TCS LSI microscope was used, with 5X (optical zoom 20X), numerical aperture 0.5. Confocal Imaging 405 nm, 488 nm and 532 nm excitation wavelengths were used for nuclei staining (Hoechst), Alexa Fluor 488-llabeled bacteria and Alexa Fluor 568-labelled phalloidin, and signals were detected with an ultra-high dynamic PMT spectral detector (430-750nm). Emitted fluorescence was split with four dichroic mirrors (QD405nm, 488nm 561nm and 635nm). Images (1024x1024) acquired with a 0.7-μm Z step were smoothed by median filtering at kernel size 3x3 pixels. ***Z*** projection of intestinal epithelium were performed using ImageJ software (NIH). Villi and crypt were visualized by Hoechst and phalloidin signals.

For quantification of tissue associated bacterial signals stacks Z step were smoothed by median filtering at kernel size 3x3 pixels. Nuin PBSmber of positive spots/1,000 μm^2^ from ileal and proximal colon and area occupied by individual spots were analyzed. Data were not normally distributed and were analyzed by non-parametric tests.

### Statistical analysis

Student’s t-test was used for pairwise comparison in most experiment. Where stated, non-parametric test were used.

## Supporting information

S1 Fig Subset of *Clostridium difficile* strains representing a wide variety of ribotypes and *C*. *difficile* genome groups. (TIF)

S2 Fig Amino acid alignment of CdeC of strain 630*erm* in members of the Peptostreptococcaceae family. **(A)** Legend of the species found to contain a homologue of CdeC. **(B)** Multiple sequence alignment was performed using local pair FLAG of MAFFT v7.294b b [46] as described in the Material and Methods section.
(TIF)

S3 Fig Amino acid alignment of CdeM of strain 630*erm* in members of the Peptostreptococcaceae family. **(A)** Legend of the species found to contain a homologue of CdeM. **(B)** Multiple sequence alignment was performed using localpair FLAG of MAFFT v7.294b b [46] as described in the Material and Methods section.
(TIF)

S4 Fig Phylogenetic tree of *cdeC* and *cdeM* in members of Peptostreptococcaceae. The phylogenetic trees were calculated using distance-based UPGMA model as described in the Material and Methods section for CdeC **(A)** and CdeM **(B)**.
(TIF)

S5 Fig Phylogenetic tree of *cdeC* in members of Clostridiaceae. The phylogenetic trees were calculated using distance-based UPGMA model as described in the Material and Methods section.
(TIF)

S6 Fig Amino acid alignment of CdeC of strain 630*erm* in members of the Clostridiaceae family. **(A)** Legend of the species found to contain a homologue of CdeC. **(B)** Multiple sequence alignment was performed using localpair FLAG of MAFFT v7.294b b [46] as described in the Material and Methods section.
(TIF)

S7 Fig Web logo of CdeC in Peptostreptococcaceae and Clostridiaceae. Sequence motifs were analyzed using SeqLogoV2.0 as described in the Material and Methods section.
(TIF)

S8 Fig Construction of *cdeC* and *cdeM C*. *difficile* mutants in 630*erm* strain. **(A, B)** Schematic representation of the intron-insertion site in *cdeC* **(A)** and *cdeM* **(B)** ORFs. Screening for intron insertion into *cdeC* **(A)** and *cdeM* **(B)** in lincomycin resistance colonies of *C*. *difficile* by PCR. The numbers at the top of the gel are the bacterial colony numbers of lincomycin-resistant trans conjugants; colonies 2, 4 and 8 (showing a ~2.8-kb band) were used for subsequent characterization for *cdeC* **(A)**, while and 2, 3 and 4 (showing a ~2.4-kb band) were used for subsequent characterization of *cdeM* **(B)**. **(C)** The site of insertion of the intron::*ermB* sequence is indicated in the bottom of panel **A** and **B**. The mutant allele is shown with an intron designed ClosTron insertion site and the number is showing the number of bp downstream of the ORF´s initiation coding site and the letter "a" indicates insertion in the antisense strand. The 45-bp retargeted sequence produces during the http://www.clostron.com algorithm and used for mutant construction. The intron insertion site within the 45-mer target sequence is shown.
(TIF)

S9 Fig Schematic representation of intestinal loop model of *C*. *difficile* infection and sample processing. C57BL/6 mice were anesthetized with isoflurane, intestinal loops of approximately 1.5 cm of the small intestine and colon were prepared and injected with 3.3x10^8^ spores per cm. Intestinal loops were incubated for 5 h prior to removal and further analyzed for immunofluorescence of *C*. *difficile* spores (for details, see Material and Methods). This scheme shows the general progression for this preparation.
(TIF)

S10 Fig Germination of *C*. *difficile* wild-type, *cdeC* and *cdeM*. **(A,B)** Germination of *C difficile* spores of wild-type, *cdeC* and *cdeM* mutant strains and their respective strains complemented with wild-type, *cdeC* and *cdeM* genes, respectively, were assessed for germination with 10 mM sodium taurocholate. For clarity, panel **A** shows spore germination of wild-type, *cdeC* and *cdeC*/*cdeC* spores. The same data for wild-type spores is presented in panels **A** and **B** for representative purposes. **(C,D)** Germination of *C*. *difficile* spores of wild-type, *cdeC* and *cdeM* mutant strains and their respective strains complemented with wild-type, *cdeC* and *cdeM* genes, respectively, were assessed for germination with phosphate buffer saline. For clarity, panel **C** shows spore germination of wild-type, *cdeC* and *cdeC*/*cdeC* spores. The same data for wild-type spores is presented in panels **C** and **D** for representative purposes.
(TIF)

S11 Fig Effect of inactivation of *cdeC* and *cdeM* in cytotoxicity in Vero cells. Cytotoxicity of supernatant of wild-type, *cdeC* and *cdeM* and their complemented strains was assessed by infecting monolayers of Vero cells and incubated for 24 h and toxin-end titer as described in Material and Methods.
(TIF)

S12 Fig Supplementary information of the mouse model of recurrent infection. These are supplementary figures from the experiment detailed in Figure 11. (**A)** relative weight during the first episode. **(B)** animals, were treated with vancomycin for 5 days to induce CDI-R and animals were monitored during CDI-R for relative weight during recurrence. **(C)** Fecal *C*. *difficile* spore shedding during initiation of CDI. **(D)** Fecal *C*. *difficile* spore shedding during recurrence of CDI. **(E)** Detection of *C*. *difficile* vegetative cells with serum of infected animals. Error bars are standard error of the mean. n.s., is no significance.
(TIF)

S13 Fig Effect of the mutation in *cdeC* on the relative abundance of selected outer surface spore proteins. The coat and exosporium extracts of 4x10^7^ spores (OD_600 nm_ = 0.2) of: **(A)** 630*erm* and *cdeC* strains carrying a *bclA1*-FLAG fusion (pDP361); **(B)** 630*erm* and *cdeC* strains carrying a *bclA2*-FLAG fusion (pDP369); **(C)** 630*erm* and *cdeC* strains carrying a *bclA3*-FLAG fusion (pDP363); **(D)** 630*erm* and *cdeC* strains carrying a *cdeA*-FLAG fusion (pDP365); **(E)**, 630*erm* and *cdeC* strains carrying a *cdeB*-FLAG fusion (pDP366); **(F)** 630*erm* and *cdeC* strains carrying a *cdeM*-FLAG fusion (pDP360); **(G)** 630*erm* and *cdeC* strains carrying a *cotA*-FLAG fusion (pDP364); **(H)** 630*erm* and *cdeC* strains carrying a *cotB*-FLAG fusion (pDP350), were extracted with SDS-PAGE loading buffer, electrophoresed and analyzed by Western blot as described in the Methods and Methods section. All experiments were done three independent times. The data shown in the graphs represent the average ± the standard error of the relative abundance. Asterisks (*) denote statistical difference at *P* < 0.05.
(TIF)

S14 Fig Effect of the mutation in *cdeM* on the relative abundance of selected outer surface spore proteins. The coat and exosporium extracts of 4x10^7^ spores of: **(A)** 630*erm* and *cdeM* strains carrying a *bclA1*-FLAG fusion (pDP361); **(B)** 630*erm* and *cdeM* strains carrying a *bclA2*-FLAG fusion (pDP369); **(C)** 630*erm* and *cdeM* strains carrying a *bclA3*-FLAG fusion (pDP363); **(D)** 630*erm* and *cdeM* strains carrying a *cdeA*-FLAG fusion (pDP365); **(E)** 630*erm* and *cdeM* strains carrying a *cdeB*-FLAG fusion (pDP366); **(F)** 630*erm* and *cdeM* strains carrying a *cdeC*-FLAG fusion (pDP345); **(G)** 630*erm* and *cdeM* strains carrying a *cotA*-FLAG fusion (pDP364); **(H)**, 630*erm* and *cdeM* strains carrying a *cotB*-FLAG fusion (pDP350), were extracted with SDS-PAGE loading buffer, electrophoresed and analyzed by Western blot as described in the Material and Methods section. All experiments were done three independent times. The data shown in the graphs represent the average ± the standard error of the relative abundance. Asterisks (*) denote statistical difference at *P* < 0.05.
(TIF)

S15 Fig Linearity of anti-flag antibody. Densitometric analysis of western blots of wild-type, *cdeC* and *cdeM* mutant strains carrying BclA1-FLAG fusions (A,B) and CdeA-FLAG fusions (C,D) were done for various concentrations of the anti-FLAG antibody: 1:1000, 1:5000 and 1:10000. Data shows that at 1:1000 and 1:5000 the differences in band intensity between strains are maintained.
(TIF)

S1 Table Bacterial strains and plasmids used. (DOCX)

S2 Table *Clostridium difficile* strains used for alignment. (XLSX)

S3 Table Peptostreptococcaceae gene conservation and loci of *cdeC*. (XLSX)

S4 Table Peptostreptococcaceae gene conservation and loci of *cdeM*. (XLSX)

S5 Table Clostridiaceae gene conservation and loci of *cdeC*. (XLSX)
