# Supplementary figures and images for: Clostridium difficile exosporium cysteine-rich proteins are essential for the morphogenesis of the exosporium layer, spore resistance, and affect C. difficile pathogenesis
Source: PLoS Pathog. 2018 Aug 8;14(8):e1007199. doi: 10.1371/journal.ppat.1007199 (PMC6101409; doi:10.1371/journal.ppat.1007199)

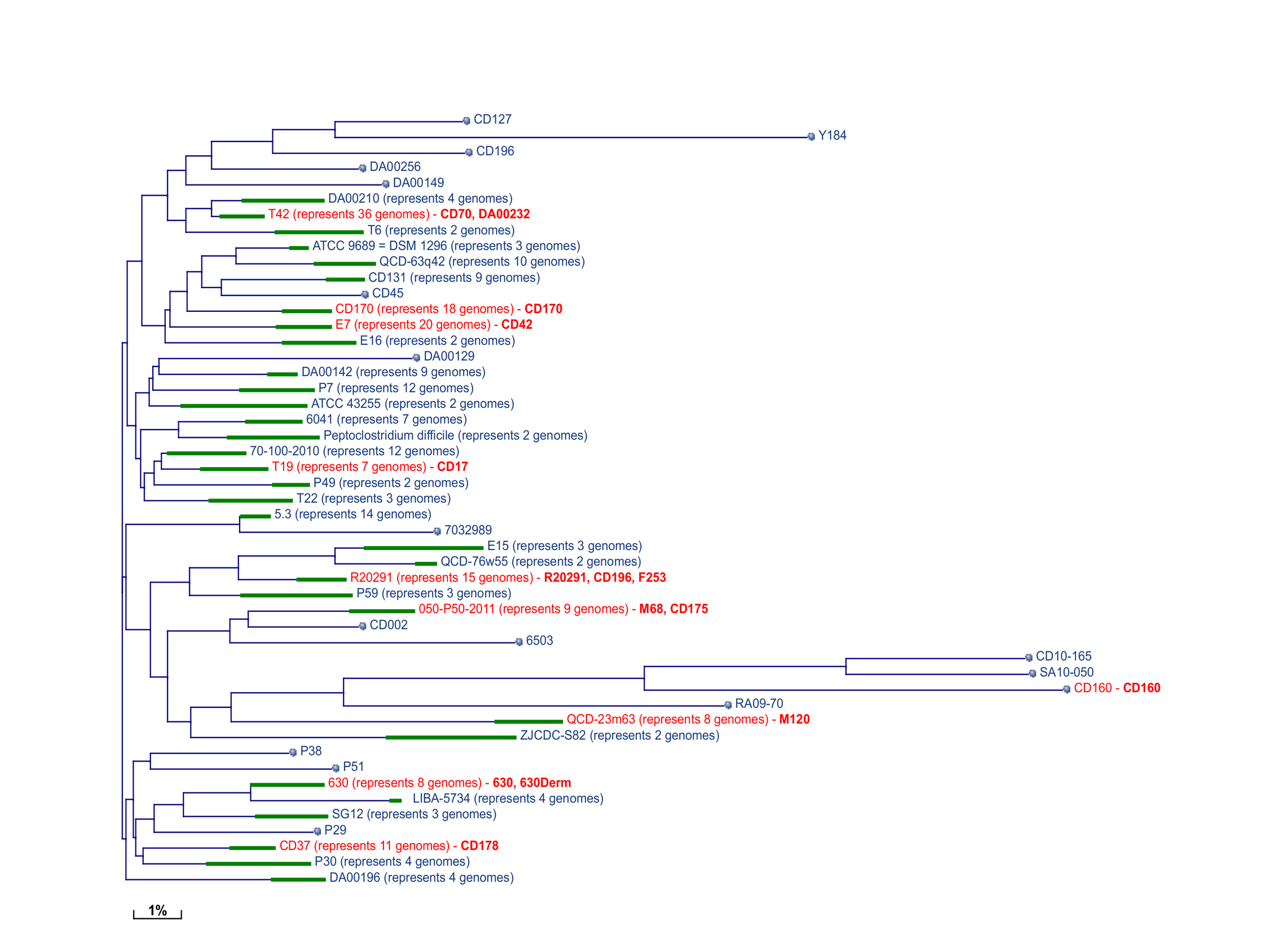

Supplement: S1 Fig — (TIF) [file ppat.1007199.s001.tif]

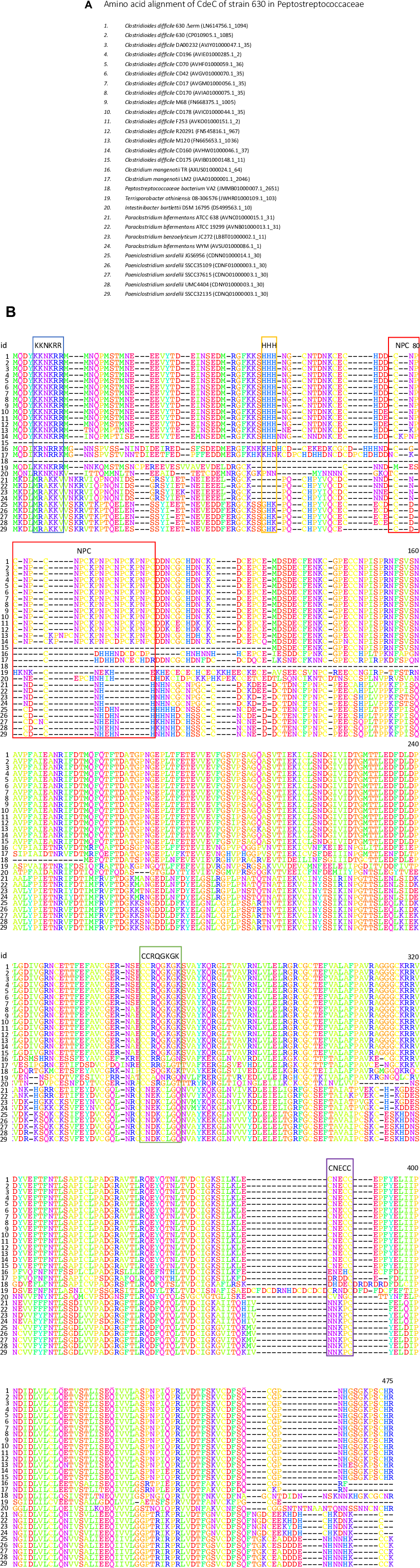

Supplement: S2 Fig — (A) Legend of the species found to contain a homologue of CdeC. (B) Multiple sequence alignment was performed using local pair FLAG of MAFFT v7.294b b [46] as described in the Material and Methods section. (TIF) [file ppat.1007199.s002.tif]

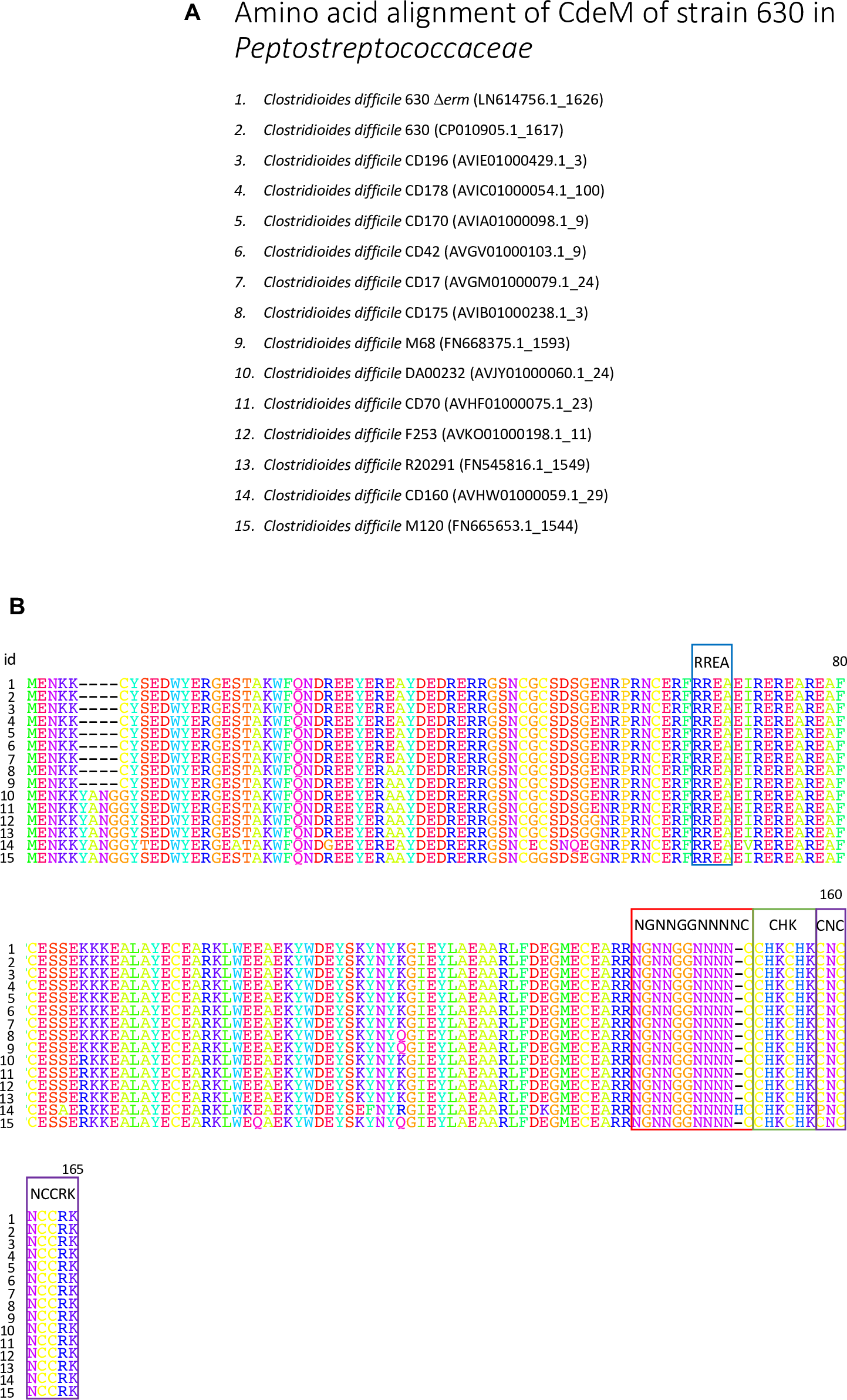

Supplement: S3 Fig — (A) Legend of the species found to contain a homologue of CdeM. (B) Multiple sequence alignment was performed using localpair FLAG of MAFFT v7.294b b [46] as described in the Material and Methods section. (TIF) [file ppat.1007199.s003.tif]

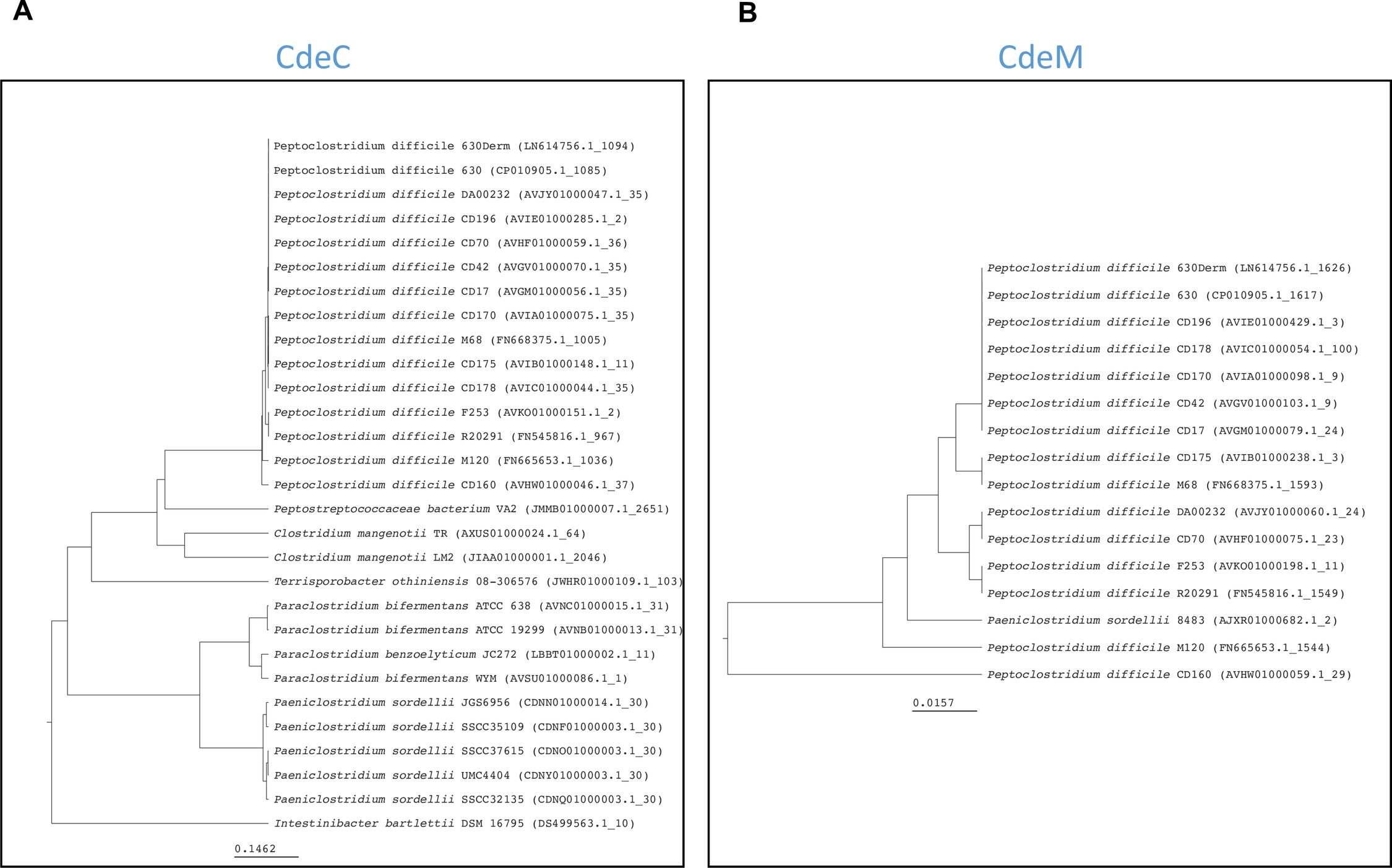

Supplement: S4 Fig — The phylogenetic trees were calculated using distance-based UPGMA model as described in the Material and Methods section for CdeC (A) and CdeM (B). (TIF) [file ppat.1007199.s004.tif]

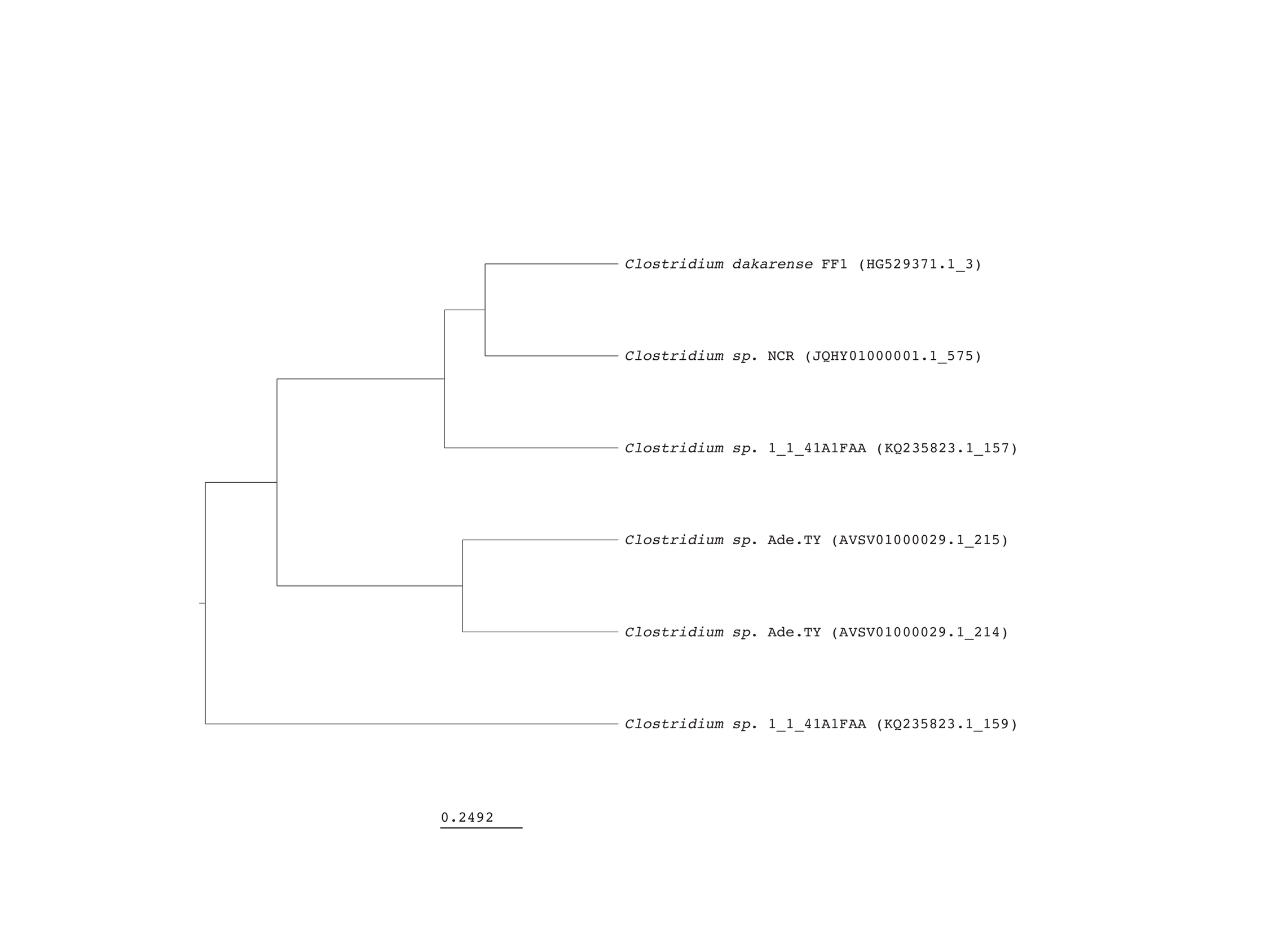

Supplement: S5 Fig — The phylogenetic trees were calculated using distance-based UPGMA model as described in the Material and Methods section. (TIF) [file ppat.1007199.s005.tif]

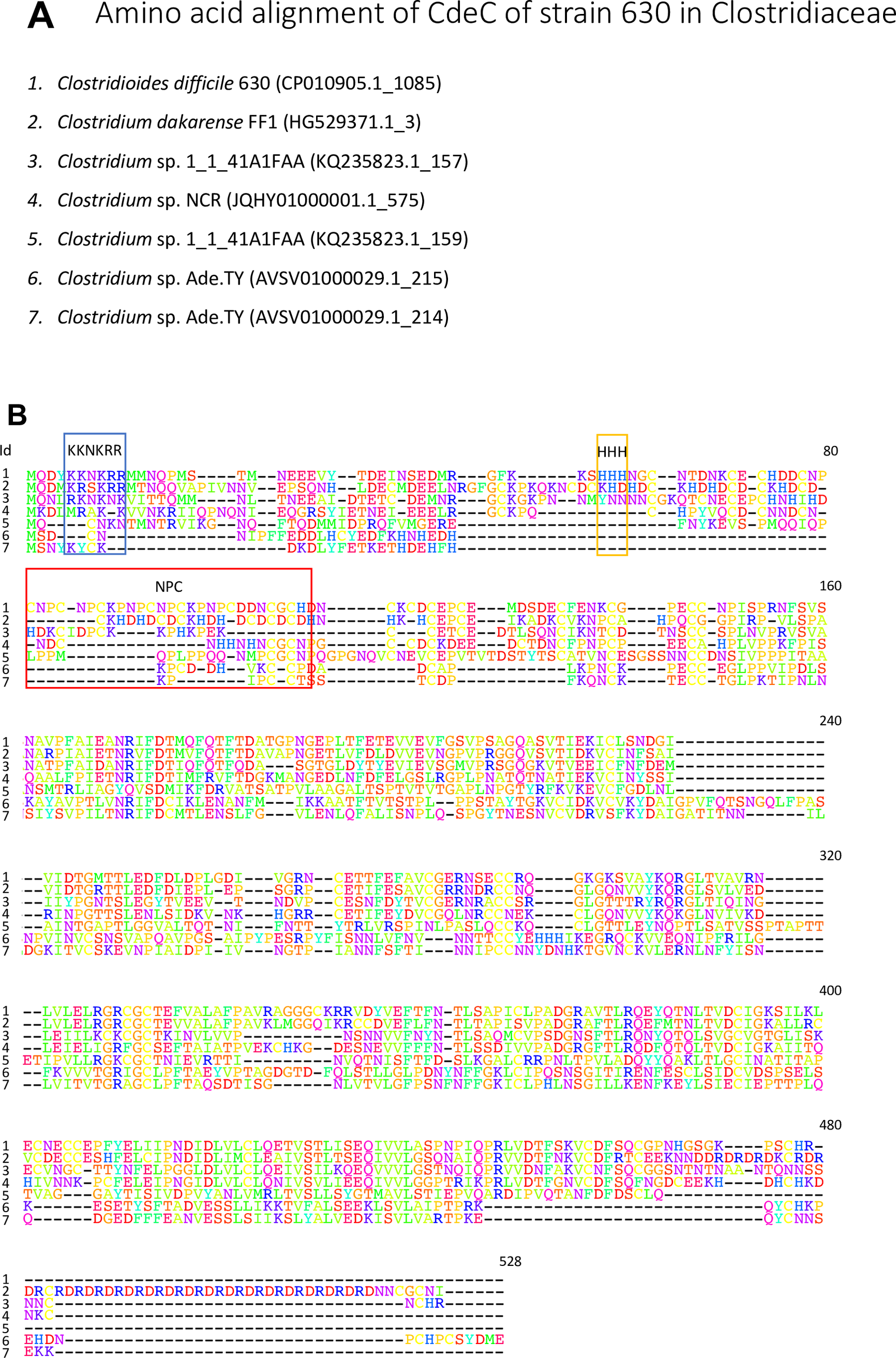

Supplement: S6 Fig — (A) Legend of the species found to contain a homologue of CdeC. (B) Multiple sequence alignment was performed using localpair FLAG of MAFFT v7.294b b [46] as described in the Material and Methods section. (TIF) [file ppat.1007199.s006.tif]

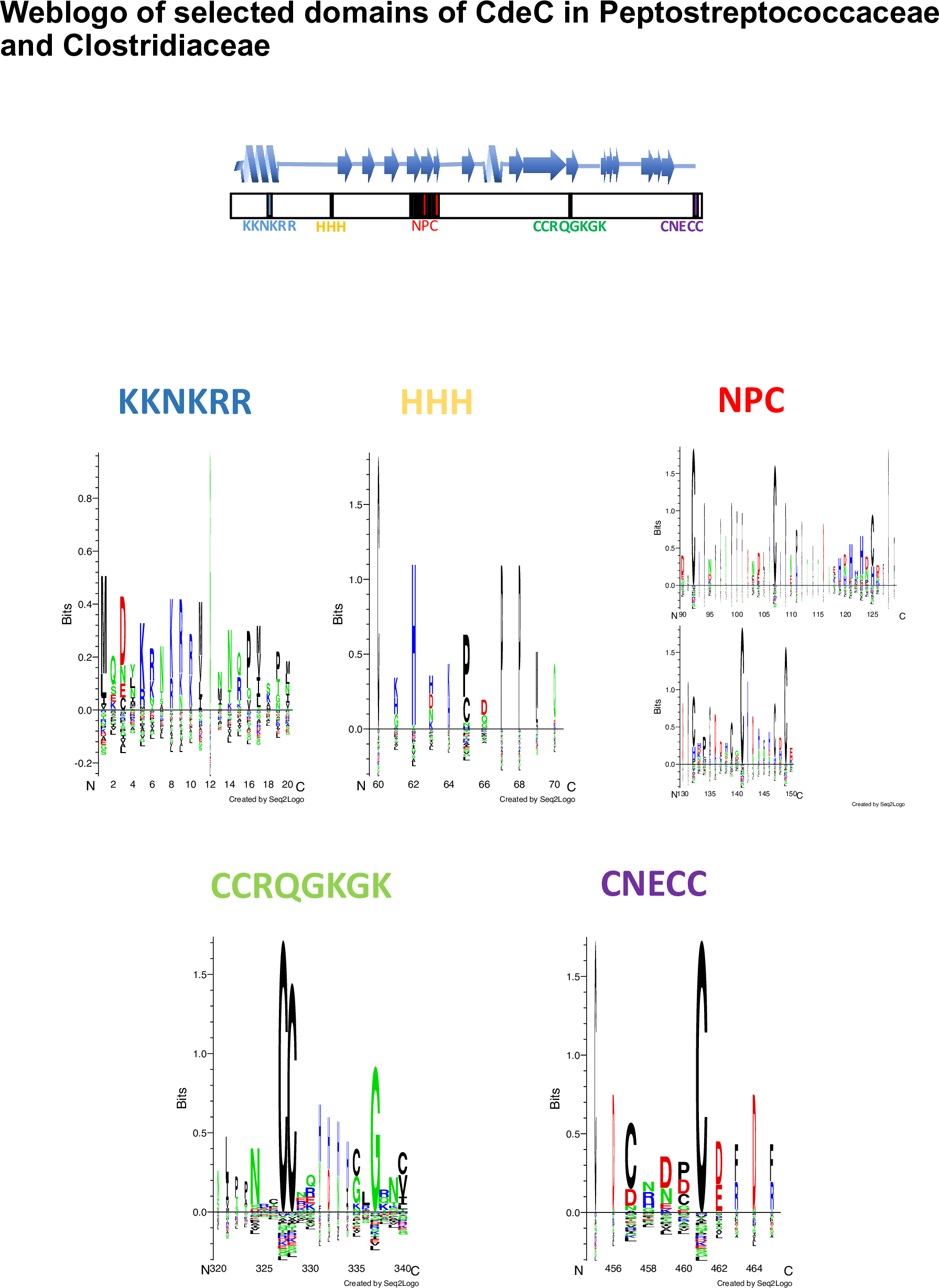

Supplement: S7 Fig — Sequence motifs were analyzed using SeqLogoV2.0 as described in the Material and Methods section. (TIF) [file ppat.1007199.s007.tif]

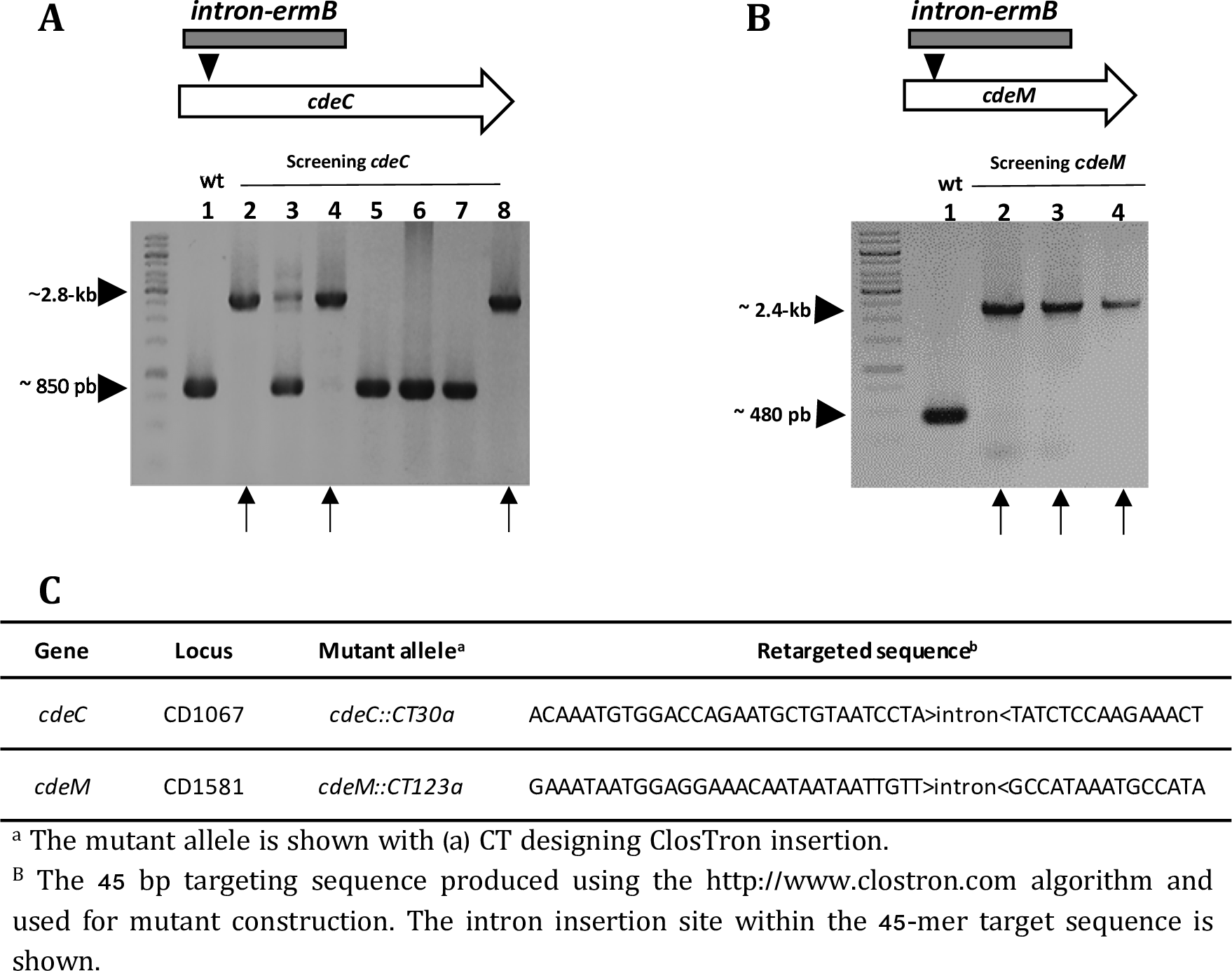

Supplement: S8 Fig — (A, B) Schematic representation of the intron-insertion site in cdeC (A) and cdeM (B) ORFs. Screening for intron insertion into cdeC (A) and cdeM (B) in lincomycin resistance colonies of C. difficile by PCR. The numbers at the top of the gel are the bacterial colony numbers of lincomycin-resistant trans conjugants; colonies 2, 4 and 8 (showing a ~2.8-kb band) were used for subsequent characterization for cdeC (A), while and 2, 3 and 4 (showing a ~2.4-kb band) were used for subsequent characterization of cdeM (B). (C) The site of insertion of the intron::ermB sequence is indicated in the bottom of panel A and B. The mutant allele is shown with an intron designed ClosTron insertion site and the number is showing the number of bp downstream of the ORF´s initiation coding site and the letter "a" indicates insertion in the antisense strand. The 45-bp retargeted sequence produces during the http://www.clostron.com algorithm and used for mutant construction. The intron insertion site within the 45-mer target sequence is shown. (TIF) [file ppat.1007199.s008.tif]

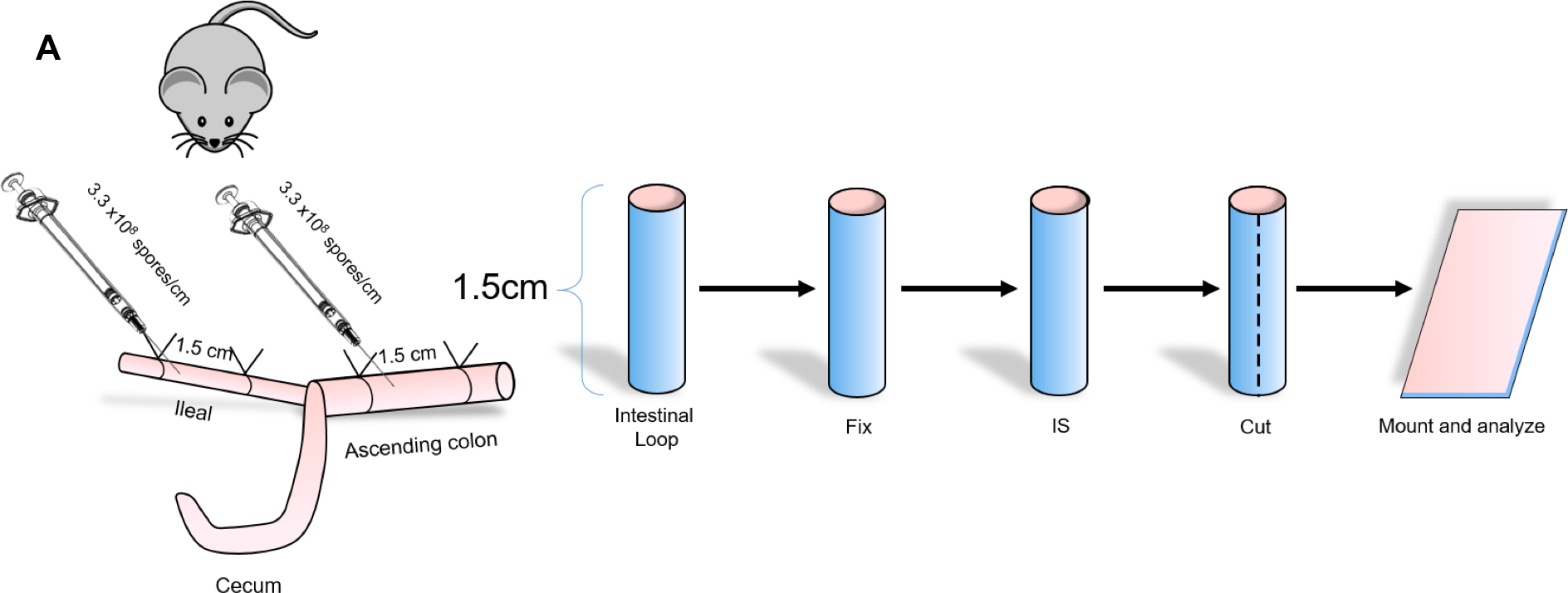

Supplement: S9 Fig — C57BL/6 mice were anesthetized with isoflurane, intestinal loops of approximately 1.5 cm of the small intestine and colon were prepared and injected with 3.3x10^8^ spores per cm. Intestinal loops were incubated for 5 h prior to removal and further analyzed for immunofluorescence of C. difficile spores (for details, see Material and Methods). This scheme shows the general progression for this preparation. (TIF) [file ppat.1007199.s009.tif]

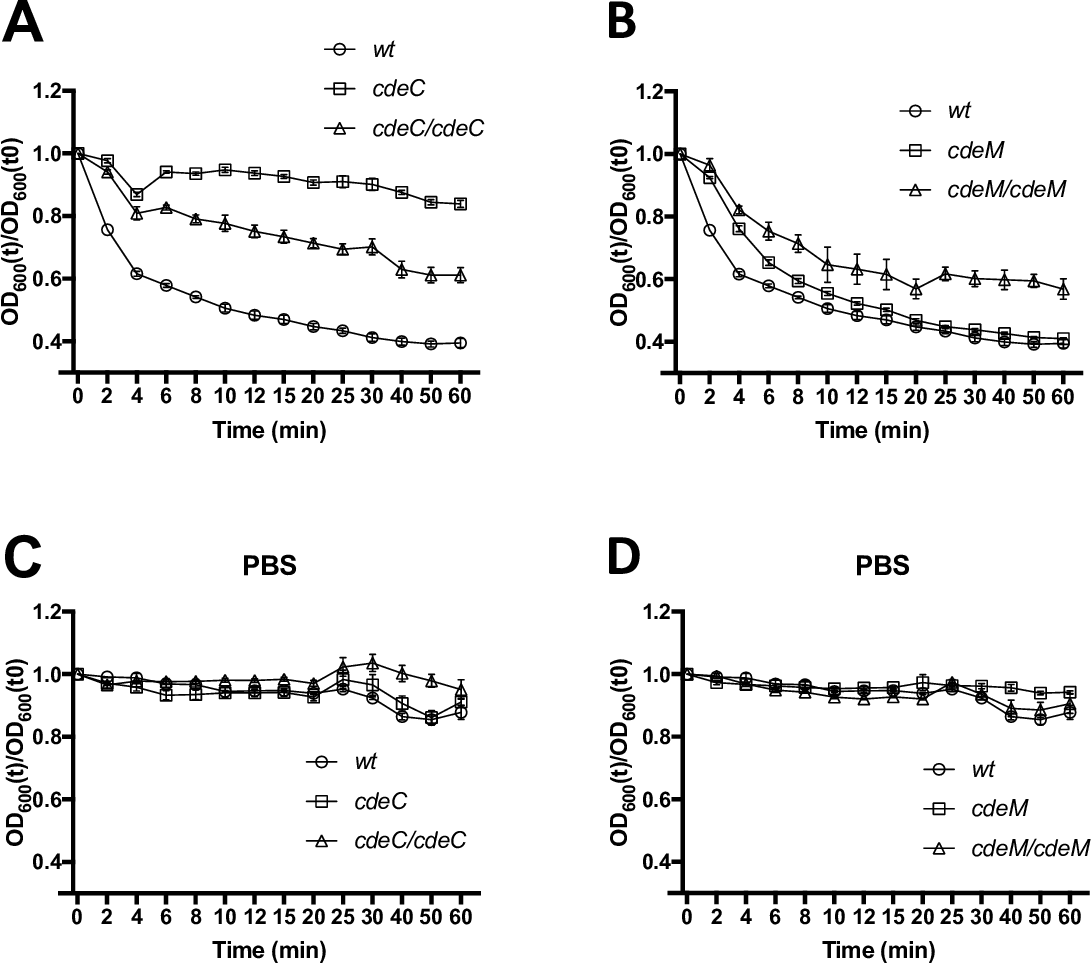

Supplement: S10 Fig — (A,B) Germination of C difficile spores of wild-type, cdeC and cdeM mutant strains and their respective strains complemented with wild-type, cdeC and cdeM genes, respectively, were assessed for germination with 10 mM sodium taurocholate. For clarity, panel A shows spore germination of wild-type, cdeC and cdeC/cdeC spores. The same data for wild-type spores is presented in panels A and B for representative purposes. (C,D) Germination of C. difficile spores of wild-type, cdeC and cdeM mutant strains and their respective strains complemented with wild-type, cdeC and cdeM genes, respectively, were assessed for germination with phosphate buffer saline. For clarity, panel C shows spore germination of wild-type, cdeC and cdeC/cdeC spores. The same data for wild-type spores is presented in panels C and D for representative purposes. (TIF) [file ppat.1007199.s010.tif]

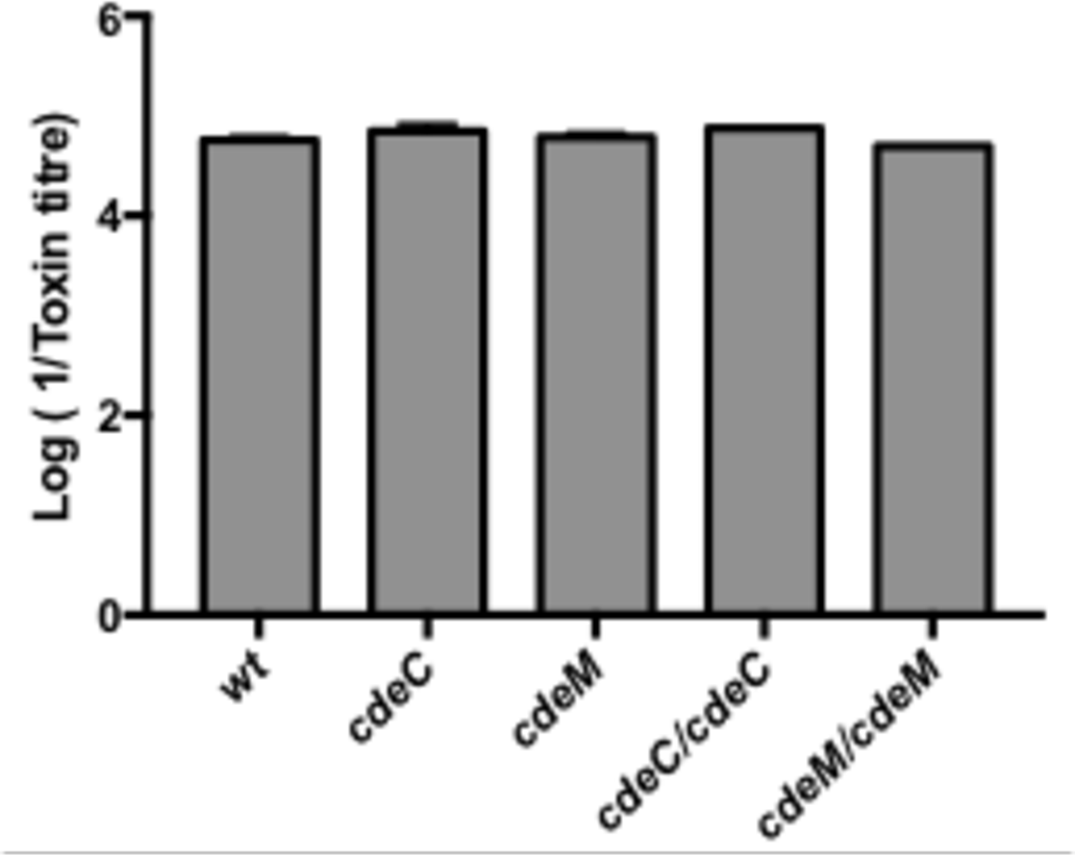

Supplement: S11 Fig — Cytotoxicity of supernatant of wild-type, cdeC and cdeM and their complemented strains was assessed by infecting monolayers of Vero cells and incubated for 24 h and toxin-end titer as described in Material and Methods. (TIF) [file ppat.1007199.s011.tif]

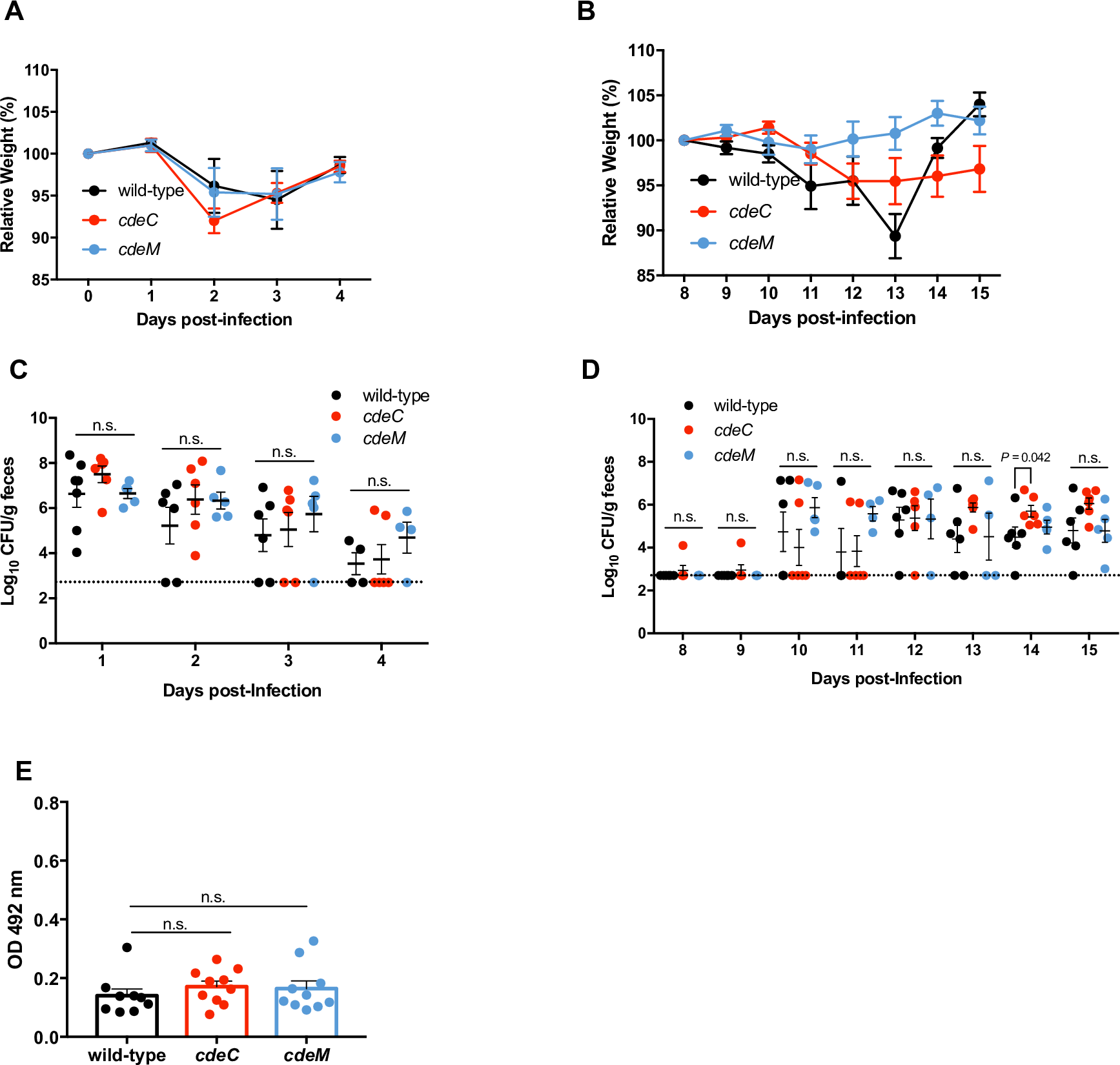

Supplement: S12 Fig — These are supplementary figures from the experiment detailed in Figure 11. (A) relative weight during the first episode. (B) animals, were treated with vancomycin for 5 days to induce CDI-R and animals were monitored during CDI-R for relative weight during recurrence. (C) Fecal C. difficile spore shedding during initiation of CDI. (D) Fecal C. difficile spore shedding during recurrence of CDI. (E) Detection of C. difficile vegetative cells with serum of infected animals. Error bars are standard error of the mean. n.s., is no significance. (TIF) [file ppat.1007199.s012.tif]

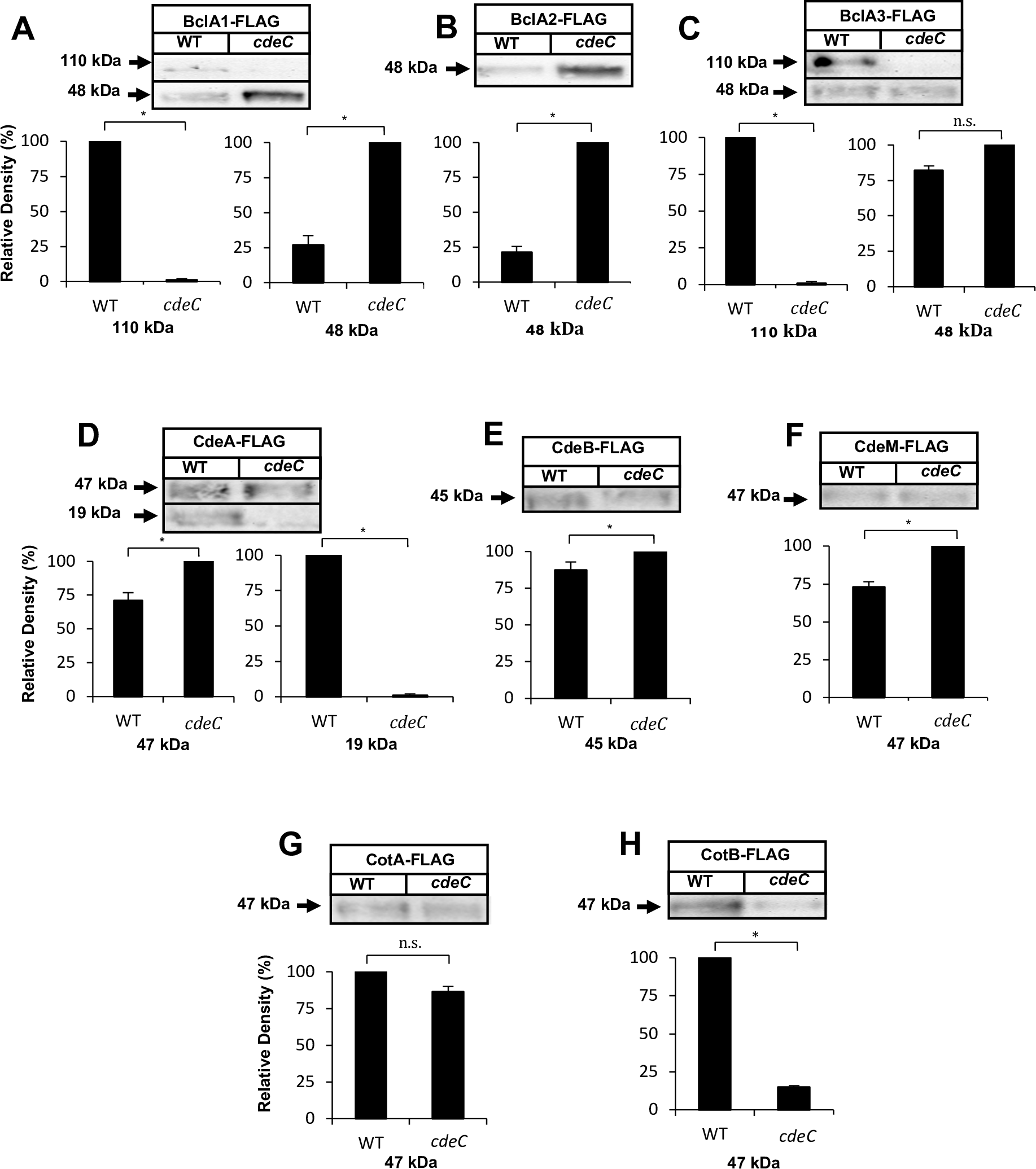

Supplement: S13 Fig — The coat and exosporium extracts of 4x10^7^ spores (OD600 nm = 0.2) of: (A) 630erm and cdeC strains carrying a bclA1-FLAG fusion (pDP361); (B) 630erm and cdeC strains carrying a bclA2-FLAG fusion (pDP369); (C) 630erm and cdeC strains carrying a bclA3-FLAG fusion (pDP363); (D) 630erm and cdeC strains carrying a cdeA-FLAG fusion (pDP365); (E), 630erm and cdeC strains carrying a cdeB-FLAG fusion (pDP366); (F) 630erm and cdeC strains carrying a cdeM-FLAG fusion (pDP360); (G) 630erm and cdeC strains carrying a cotA-FLAG fusion (pDP364); (H) 630erm and cdeC strains carrying a cotB-FLAG fusion (pDP350), were extracted with SDS-PAGE loading buffer, electrophoresed and analyzed by Western blot as described in the Methods and Methods section. All experiments were done three independent times. The data shown in the graphs represent the average ± the standard error of the relative abundance. Asterisks (*) denote statistical difference at P < 0.05. (TIF) [file ppat.1007199.s013.tif]

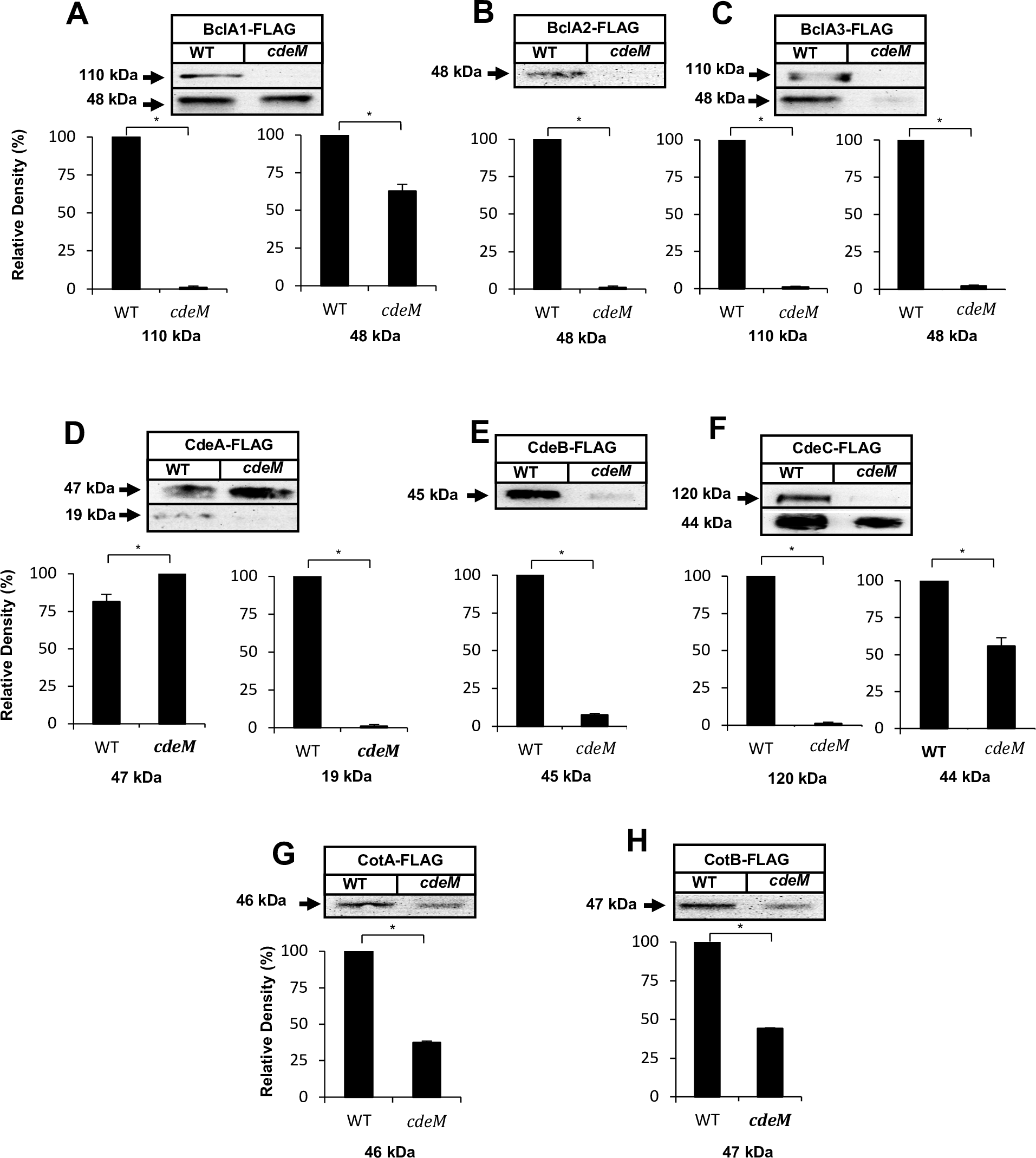

Supplement: S14 Fig — The coat and exosporium extracts of 4x10^7^ spores of: (A) 630erm and cdeM strains carrying a bclA1-FLAG fusion (pDP361); (B) 630erm and cdeM strains carrying a bclA2-FLAG fusion (pDP369); (C) 630erm and cdeM strains carrying a bclA3-FLAG fusion (pDP363); (D) 630erm and cdeM strains carrying a cdeA-FLAG fusion (pDP365); (E) 630erm and cdeM strains carrying a cdeB-FLAG fusion (pDP366); (F) 630erm and cdeM strains carrying a cdeC-FLAG fusion (pDP345); (G) 630erm and cdeM strains carrying a cotA-FLAG fusion (pDP364); (H), 630erm and cdeM strains carrying a cotB-FLAG fusion (pDP350), were extracted with SDS-PAGE loading buffer, electrophoresed and analyzed by Western blot as described in the Material and Methods section. All experiments were done three independent times. The data shown in the graphs represent the average ± the standard error of the relative abundance. Asterisks (*) denote statistical difference at P < 0.05. (TIF) [file ppat.1007199.s014.tif]

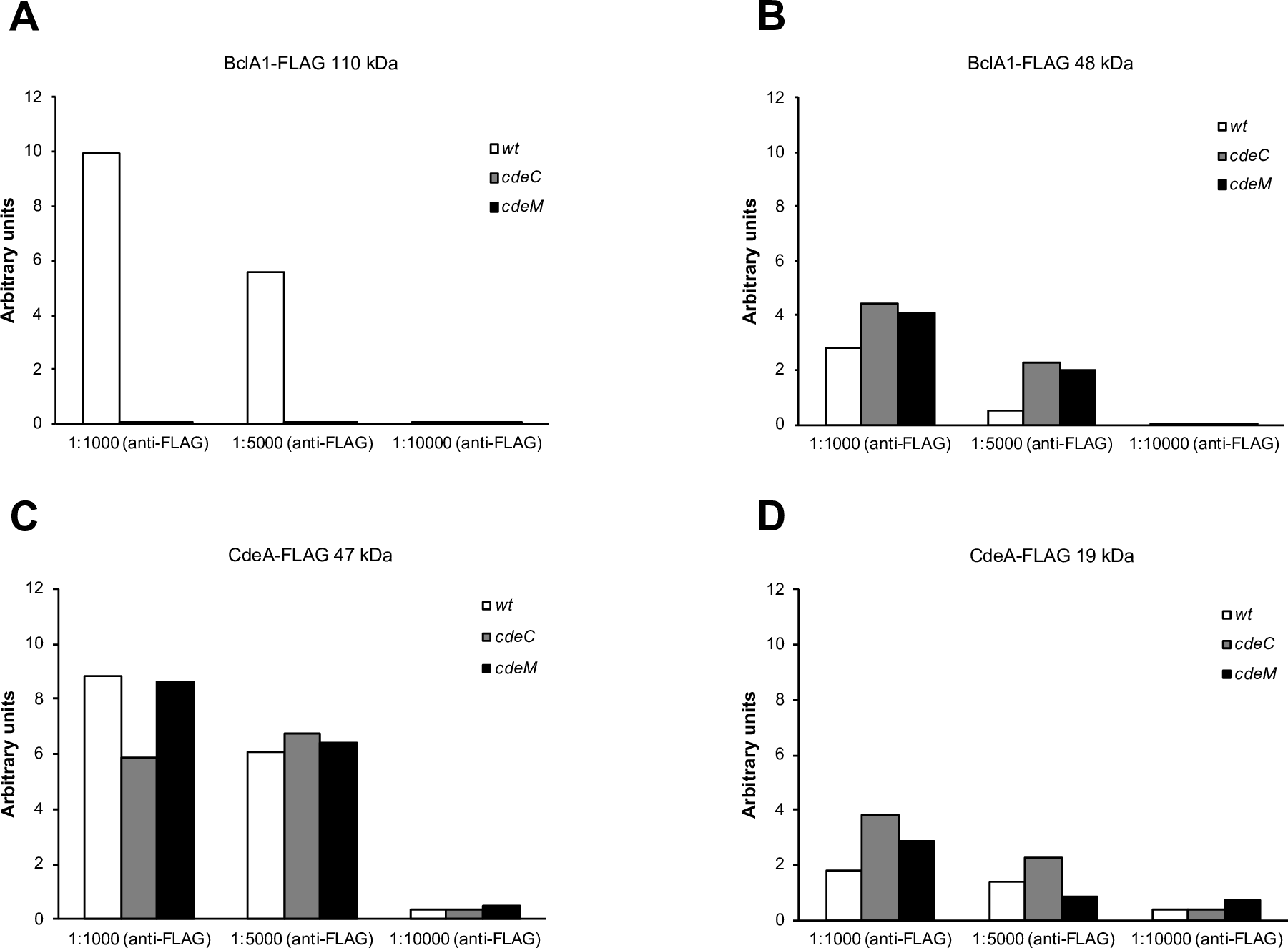

Supplement: S15 Fig — Densitometric analysis of western blots of wild-type, cdeC and cdeM mutant strains carrying BclA1-FLAG fusions (A,B) and CdeA-FLAG fusions (C,D) were done for various concentrations of the anti-FLAG antibody: 1:1000, 1:5000 and 1:10000. Data shows that at 1:1000 and 1:5000 the differences in band intensity between strains are maintained. (TIF) [file ppat.1007199.s015.tif]
