# Supplementary material for: Clostridium difficile exosporium cysteine-rich proteins are essential for the morphogenesis of the exosporium layer, spore resistance, and affect C. difficile pathogenesis
Source: PLoS Pathog. 2018 Aug 8;14(8):e1007199. doi: 10.1371/journal.ppat.1007199 (PMC6101409; doi:10.1371/journal.ppat.1007199)
Supplement: S1 Table — (DOCX) [file ppat.1007199.s016.docx]

**Supplementary Tables**

| TABLE S1. Bacterial strains and plasmids used | | |
| --- | --- | --- |
| Strain or Plasmid | Relevant characteristic | Source/Reference |
| ***C. difficile*** |  |  |
| 630Δ*ermB* | An erythromycin sensitive derivative of *C. difficile* strain 630 | [1] |
| 630Δ*ermB*(pDP345) | 630∆*ermB* carrying *cdeC*-FLAG fusion | [2] |
| 630Δ*ermB*(pDP350) | 630∆*ermB* carrying *cotB*-FLAG fusion | [2] |
| 630Δ*ermB*(pDP360) | 630∆*ermB* carrying *cdeM*-FLAG fusion | [2] |
| 630Δ*ermB*(pDP361) | 630∆*ermB* carrying *bclA1*-FLAG fusion | [2] |
| 630Δ*ermB*(pDP363) | 630∆*ermB* carrying *bclA3*-FLAG fusion | [2] |
| 630Δ*ermB*(pDP364) | 630∆*ermB* carrying *cotA*-FLAG fusion | [2] |
| 630Δ*ermB*(pDP365) | 630∆*ermB* carrying *cdeA*-FLAG fusion | [2] |
| 630Δ*ermB*(pDP366) | 630∆*ermB* carrying *cdeB*-FLAG fusion | [2] |
| 630Δ*ermB*(pDP369) | 630∆*ermB* carrying *bclA2*-FLAG fusion | [2] |
| 630 Δ*ermB cdeC* | 630 Δ*ermB cdeC*::intron *ermB* | This work |
| 630 Δ*ermB cdeM* | 630 Δ*ermB cdeM*::intron *ermB* | This work |
| 630Δ*ermB* *cdeC* (pDP345) | 630∆*ermB* *cdeC::*intron carrying *cdeC*-FLAG fusion | This work |
| 630Δ*ermB cdeC* (pDP350) | 630∆*ermB* *cdeC::*intron carrying *cotB*-FLAG fusion | This work |
| 630Δ*ermB cdeC* (pDP360) | 630∆*ermB* *cdeC::*intron carrying *cdeM*-FLAG fusion | This work |
| 630Δ*ermB cdeC* (pDP361) | 630∆*ermB* *cdeC::*intron carrying *bclA1*-FLAG fusion | This work |
| 630Δ*ermB cdeC* (pDP363) | 630∆*ermB* *cdeC::*intron carrying *bclA3*-FLAG fusion | This work |
| 630Δ*ermB cdeC* (pDP364) | 630∆*ermB* *cdeC::*intron carrying *cotA*-FLAG fusion | This work |
| 630Δ*ermB cdeC* (pDP365) | 630∆*ermB* *cdeC::*intron carrying *cdeA*-FLAG fusion | This work |
| 630Δ*ermB cdeC* (pDP366) | 630∆*ermB* *cdeC::*intron carrying *cdeB*-FLAG fusion | This work |
| 630Δ*ermB cdeC* (pDP369) | 630∆*ermB* *cdeC::*intron carrying *bclA2*-FLAG fusion | This work |
| 630Δ*ermB cdeM* (pDP345) | 630∆*ermB* *cdeM::*intron carrying *cdeC*-FLAG fusion | This work |
| 630Δ*ermB cdeM* (pDP350) | 630∆*ermB* *cdeM::*intron carrying *cotB*-FLAG fusion | This work |
| 630Δ*ermB cdeM* (pDP360) | 630∆*ermB* *cdeM::*intron carrying *cdeM*-FLAG fusion | This work |
| 630Δ*ermB cdeM* (pDP361) | 630∆*ermB* *cdeM::*intron carrying *bclA1*-FLAG fusion | This work |
| 630Δ*ermB cdeM* (pDP363) | 630∆*ermB* *cdeM::*intron carrying *bclA3*-FLAG fusion | This work |
| 630Δ*ermB cdeM* (pDP364) | 630∆*ermB* *cdeM::*intron carrying *cotA*-FLAG fusion | This work |
| 630Δ*ermB cdeM* (pDP365) | 630∆*ermB* *cdeM::*intron carrying *cdeA*-FLAG fusion | This work |
| 630Δ*ermB cdeM* (pDP366) | 630∆*ermB* *cdeM::*intron carrying *cdeB*-FLAG fusion | This work |
| 630Δ*ermB cdeM* (pDP369) | 630∆*ermB* *cdeM::*intron carrying *bclA2*-FLAG fusion | This work |
| ***E. coli*** |  |  |
| HB101 | F^-^ *mcrB mrr hsdS20*(r_B_^-^ m_B_^-^) *recA13 leuB6 ara-14 proA2 lacY1 galK2 xyl-5 mtl-1 rpsL20* | [3] |
| BL21 λ(DE3)-RIL | *E. coli* B Fe *ompT* *hsdS*(rB- mB-) *dcm* + Tet^r^ *gal* λ(DE3) *endA* Hte [*argU* *ileY* *leuW* Cam^r^] | [4] |
| HB101(pRK24,pDP306) | HB101 containing pRK24 and pDP306 | This study |
| HB101(pRK24,pDP345) | HB101 containing pRK24 and pDP345 | This study |
| HB101(pRK24,pDP350) | HB101 containing pRK24 and pDP350 | [2] |
| HB101(pRK24,pDP360) | HB101 containing pRK24 and pDP360 | [2] |
| HB101(pRK24,pDP361) | HB101 containing pRK24 and pDP361 | [2] |
| HB101(pRK24,pDP363) | HB101 containing pRK24 and pDP363 | [2] |
| HB101(pRK24,pDP364) | HB101 containing pRK24 and pDP364 | [2] |
| HB101(pRK24,pDP365) | HB101 containing pRK24 and pDP365 | [2] |
| HB101(pRK24,pDP366) | HB101 containing pRK24 and pDP366 | [2] |
| HB101(pRK24,pDP369) | HB101 containing pRK24 and pDP369 | [2] |
| HB101(pRK24,pDP370) | HB101 containing pRK24 and pDP370 | This study |
|  |  |  |
|  |  |  |
| ***Plasmids*** |  |  |
| pMTL82151 | *E. coli*-*C. difficile* shuttle vector | [5] |
| pRK24 | Tra^+^, Mob^+^; *bla*, *tet*^r^ | [6] |
| pDP306 | pMTL007C-E2::Cdi-*cdeC*-30a | [7] |
| pDP345 | pMTL82151 carrying *cdeC*_630_ labeled at the C-terminal with FLAG as a reporter tag in NdeI/HindIII sites. | [7] |
| pDP350 | pMTL82151 carrying *cotB*_630_ labeled at the C-terminal with FLAG as a reporter tag in NdeI/HindIII sites. | [2] |
| pDP360 | pMTL82151 carrying *cdeM*_630_ labeled at the C-terminal with FLAG as a reporter tag in KpnI/SalI sites. | [2] |
| pDP361 | pMTL82151 carrying *bclA1*_630_ labeled at the C-terminal with FLAG as a reporter tag in KpnI/SalI sites. | [2] |
| pDP363 | pMTL82151 carrying *bclA3*_630_ labeled at the C-terminal with FLAG as a reporter tag in KpnI/SalI sites. | [2] |
| pDP364 | pMTL82151 carrying *cotA*_630_ labeled at the C-terminal with FLAG as a reporter tag in KpnI/SalI sites. | [2] |
| pDP365 | pMTL82151 carrying *cdeA*_630_ labeled at the C-terminal with FLAG as a reporter tag in KpnI/SalI sites. | [2] |
| pDP366 | pMTL82151 carrying *cdeB*_630_ labeled at the C-terminal with FLAG as a reporter tag in KpnI/SalI sites. | [2] |
| pDP369 | pMTL82151 carrying *bclA2*_630_ labeled at the C-terminal with FLAG as a reporter tag in KpnI/SalI sites. | [2] |
| pDP370 | pMTL007C-E2::Cdi-*cdeM*-123a | This study |
|  |  |  |
|  |  |  |

**References**

1. Hussain HA, Roberts AP, Mullany P. Generation of an erythromycin-sensitive derivative of *Clostridium difficile* strain 630 (630Deltaerm) and demonstration that the conjugative transposon Tn916DeltaE enters the genome of this strain at multiple sites. J Med Microbiol. 2005;54: 137–41. doi:10.1099/jmm.0.45790-0

2. Díaz-González F, Milano M, Olguin-Araneda V, Pizarro-Cerda J, Castro-Córdova P, Tzeng S-C, et al. Protein composition of the outermost exosporium-like layer of *Clostridium difficile* 630 spores. J Proteomics. Elsevier B.V.; 2015;123: 1–13. doi:10.1016/j.jprot.2015.03.035

3. Serebrijski I, Reyes O, Leblon G. Corrected gene assignments of *Escherichia coli* pro- mutations. J Bacteriol. 1995;177: 7261–4.

4. Studier W, Moffatt B. Use of bacteriophage T7 RNA polymerase to direct selective high-level expression of cloned genes. J Mol Biol. 1986;189: 113–130.

5. Heap JT, Pennington OJ, Cartman ST, Minton NP. A modular system for *Clostridium* shuttle plasmids. J Microbiol Methods. 2009;78: 79–85. doi:10.1016/j.mimet.2009.05.004

6. Thomas CM, Smith CA. Incompatibility group P plasmids: genetics, evolution, and use in genetic manipulation. Annu Rev Microbiol. 1987;41: 77–101. doi:10.1146/annurev.mi.41.100187.000453

7. Barra-Carrasco J, Olguín-Araneda V, Plaza-Garrido A, Miranda-Cárdenas C, Cofré-Araneda G, Pizarro-Guajardo M, et al. The *Clostridium difficile* exosporium cysteine (CdeC)-rich protein is required for exosporium morphogenesis and coat assembly. J Bacteriol. 2013;195: 3863–75. doi:10.1128/JB.00369-13
